# Comparative genomics and transcriptomics of *Escherichia coli* isolates carrying virulence factors of both enteropathogenic and enterotoxigenic *E. coli*

**DOI:** 10.1038/s41598-017-03489-z

**Published:** 2017-06-14

**Authors:** Tracy H. Hazen, Jane Michalski, Qingwei Luo, Amol C. Shetty, Sean C. Daugherty, James M. Fleckenstein, David A. Rasko

**Affiliations:** 1 Institute for Genome Sciences, University of Maryland School of Medicine, Baltimore, USA; 2 Department of Microbiology and Immunology, University of Maryland School of Medicine, Baltimore, MD 21201 USA; 3 Department of Medicine, Division of Infectious Diseases, Washington University School of Medicine, Saint Louis, MO 63110 USA; 4 The Medicine Service, Veterans Affairs Medical Center, Saint Louis, MO 63110 USA

## Abstract

*Escherichia coli* that are capable of causing human disease are often classified into pathogenic variants (pathovars) based on their virulence gene content. However, disease-associated hybrid *E. coli*, containing unique combinations of multiple canonical virulence factors have also been described. Such was the case of the *E. coli* O104:H4 outbreak in 2011, which caused significant morbidity and mortality. Among the pathovars of diarrheagenic *E. coli* that cause significant human disease﻿ are the enteropathogenic *E. coli* (EPEC) and enterotoxigenic *E. coli* (ETEC). In the current study we use comparative genomics, transcriptomics, and functional studies to characterize isolates that contain virulence factors of both EPEC and ETEC. Based on phylogenomic analysis, these hybrid isolates are more genomically-related to EPEC, but appear to have acquired ETEC virulence genes. Global transcriptional analysis using RNA sequencing, demonstrated that the EPEC and ETEC virulence genes of these hybrid isolates were differentially-expressed under virulence-inducing laboratory conditions, similar to reference isolates. Immunoblot assays further verified that the virulence gene products were produced and that the T3SS effector EspB of EPEC, and heat-labile toxin of ETEC were secreted. These findings document the existence and virulence potential of an *E. coli* pathovar hybrid that blurs the distinction between *E. coli* pathovars.

## Introduction

*Escherichia coli* are a significant cause of diarrheal illness and mortality worldwide each year, especially among children in developing countries^1, 2^. In 2010, diarrheal illness caused an estimated 1.4 million deaths worldwide, which included over 120,000 deaths associated with enterotoxigenic *E. coli* (ETEC) and over 88,000 deaths associated with the enteropathogenic *E. coli* (EPEC)^2^. Both ETEC and EPEC cause significant diarrheal illness and mortality in children; predominately in the developing world^1, 3, 4^. ETEC has also been identified as a major cause of traveler’s diarrhea among adults worldwide^1, 2, 5^.

Diarrheagenic *E. coli* are currently classified into a small number of groups, based primarily on canonical virulence gene content, as belonging to a single pathogenic variant (pathovar). EPEC isolates that contain the Locus of Enterocyte Effacement (LEE) region and Bundle-Forming Pilus (BFP) are termed typical EPEC (tEPEC), while EPEC isolates that do not contain the BFP are called atypical EPEC (aEPEC)^3, 6, 7^. Meanwhile, ETEC are molecularly characterized by the presence of the heat-labile (LT) or heat-stable (ST) enterotoxins, as well as an assortment of accessory virulence factors, such as the EatA autotransporter^8, 9^. These canonical virulence factors are often encoded on plasmids or other mobile elements in the isolates from each of these pathovars^7, 10^. However, based on the identification of canonical virulence features, some clinical isolates could be classified to multiple pathovars. For example, an *E. coli* outbreak in Germany in 2011 that sickened over 3,400 people and caused 39 deaths was attributed to a hybrid pathogenic *E. coli* ^11^. The European *E. coli* O104:H4 isolate was phylogenomically most similar to a previously sequenced enteroaggregative *E. coli* (EAEC), but also contained the Shiga toxin phage that is a defining feature of Shiga-toxigenic *E. coli* (STEC), including the *E. coli* O157:H7, which are a significant cause of severe foodborne illnesses^7, 12, 13^. This European O104 outbreak demonstrated the significant impact of a novel disease-causing *E. coli* that blurs the definition between the different *E. coli* pathovars. ﻿Additional hybrid *E. coli* isolates have been described in the literature. For example, *E. coli* isolates containing combinations of EPEC/ETEC and STEC/ETEC virulence genes have been identified from humans and cattle^14, 15^. Among the previously characterized hybrid *E. coli* isolates with a combination with canonical virulence genes of other *E. coli* pathovars, were three isolates that contained the genes encoding Shiga toxin of STEC and the heat-stable enterotoxin (ST) of ETEC^14^. Another study identified an isolate that contained the LEE region of EPEC and the LT genes of ETEC^15^. These studies highlight the limitations of the simple pathovar definitions and identify that there may be a number of hybrid isolates circulating. Based on these phylogenomic comparisons, we and others have demonstrated that *E. coli* isolates with the same virulence gene content can be present in various locations within the phylogenomic framework of the species^16–22^. These phylogroups often contain isolates from only one pathovar, however it is possible that isolates from different pathovars are part of the same phylogroup as is the case for EPEC and ETEC^18, 21, 23, 24^.

In the current study, we use a combination of comparative genomics, transcriptomics, and functional characterization to describe four EPEC/ETEC hybrid isolates obtained from children in Africa that were enrolled in the Global Enteric Multicenter Study (GEMS)^1, 25^. These isolates are termed EPEC/ETEC hybrids to reflect that they contain a mixture of canonical and accessory virulence factors from both EPEC and ETEC pathovars. The comparative genomic and phylogenomic analyses demonstrate that the EPEC/ETEC hybrid isolates are genomically most related to EPEC, and appear to have acquired ETEC virulence genes via horizontal gene transfer. Furthermore, the comparative transcriptomics and functional characterization verify that the EPEC and ETEC virulence genes are transcriptionally- regulated and produced by these EPEC/ETEC hybrid isolates.

## Results

### Characterization of the EPEC/ETEC hybrid isolates

In the current study we analyzed two types of hybrid isolates that contained mixtures of virulence factors from EPEC and ETEC pathovars (Table 1). All four of the EPEC/ETEC hybrid isolates contained the LEE region, which is characteristic of all EPEC isolates (Table S1). The EatA+ EPEC/ETEC isolate 401140 also contained the BFP region and would therefore be considered tEPEC according to the traditional pathovar assignment (Table S1). In contrast, the heat labile toxin (LT) positive EPEC/ETEC isolates 102651, 102712, and 102771 did not contain the BFP region, thus they would be classified as aEPEC (Table S1). The four EPEC/ETEC hybrid isolates were obtained from children enrolled in GEMS sites in two countries (Table 1). The LT+ EPEC/ETEC hybrid isolates, 102651, 102712, and 102771, were obtained from children in The Gambia between July and September 2009 (Table 1). Meanwhile the EatA+ EPEC/ETEC isolate 401140 was obtained from a child in Kenya in June 2008 (Table 1).

**Table 1:** Characteristics of the EPEC/ETEC hybrid isolates.

Isolate ID	Virulence Content^a^	Clinical Outcome^b^	Location^c^	Date^c^	No. of Contigs	Genome Size (Mb)	Phylogroup^d^	EPEC Lineage^e^	MLST ST^f^
102651	LEE+/BFP−/LT+	non-lethal symptomatic	The Gambia	7/28/09	127	5.37	B1	EPEC7	328
102712	LEE+/BFP−/LT+	non-lethal symptomatic	The Gambia	8/17/09	139	5.19	B1	EPEC7	328
102771	LEE+/BFP−/LT+	asymptomatic	The Gambia	9/14/09	144	5.21	B1	EPEC7	328
401140	LEE+/BFP+/EatA+	lethal	Kenya	6/12/08	255	5	A	EPEC5	1788

^a^The virulence content is the pathovar-specific putative protein-encoding virulence genes identified in each of the hybrid isolates.

^b^The clinical outcome of the patient that each isolate was cultured from.

^c^The location and date of isolation of the patient samples the hybrid isolate was cultured from.

^d^The *E. coli* phylogroups are the same as those previously described (Jaureguy *et al*.^35^, Tenaillon *et al*.^36^).

^e^The EPEC phylogenomic lineages correspond with those previously described (Hazen *et al*.^16^).

^f^The multilocus sequence types were determined using the database hosted by the University of Warwick (http://mlst.warwick.ac.uk/mlst/dbs/Ecoli).

Many of the canonical virulence factors in *E. coli* are plasmid encoded^10, 26^, therefore we examined the plasmid content of all EPEC/ETEC hybrid isolates to determine if it was similar or different from the reference isolates E2348/69 (EPEC), E24377A (ETEC), and H10407 (ETEC) for the EPEC and ETEC pathovars. Characterization of the plasmid content of each isolate by gel electrophoresis demonstrated that all of the LT+ EPEC/ETEC isolates (102651, 102712, and 102771) contain a plasmid that is ~100 kb, while two of the isolates (102712 and 102771) have a second plasmid that is ~70 kb, which is absent from isolate 102651 (Fig. S1). Similarly, the EatA+ EPEC/ETEC isolate 401140 contains two large plasmids of ~100 kb and 120 kb (Fig. S1). There are additional smaller plasmids, <25 kb, in each of these EPEC/ETEC hybrid isolates, which are present in many *E. coli* isolates^27, 28^, but their role in virulence or survival has not been characterized. The number of identifiable plasmids by gel electrophoresis ranges between three and five for the EPEC/ETEC hybrid isolates (Fig. S1).

Three of the four EPEC/ETEC hybrid isolates (102651, 102712, and 102771) carry the *eltA* and *eltB* genes that encode the heat-labile toxin, which is one of the pathovar-specific features of ETEC^29^, whereas the remaining hybrid isolate (401140) contains the *eatA* gene, which is common in many ETEC^30^. The LT genes of the LT+ EPEC/ETEC hybrid isolates were most related to the plasmid-encoded type I LT^31^ rather than the phage-associated type II LT^32^. The gene of the LT+ EPEC/ETEC hybrid isolates that encodes LT subunit B (*eltB*) exhibited 100% nucleotide identity to *eltB* of the LT-encoding virulence plasmid p666 (FN649417.1) from ETEC isolate H10407. The complete coding region of LT subunit A (*eltA*) is present in the LT+ EPEC/ETEC genomes with only four nucleotide differences (99% nucleotide identity) when compared to the *eltA* gene of plasmid p666. However, a non-synonymous change (C569T) introduced a stop codon that truncated the predicted protein sequence by 69 aa.

Thus, molecular characterization of the EPEC/ETEC hybrid isolates demonstrated that they are distinct isolates, are from different patients, and contain combinations of the EPEC and ETEC virulence genes when compared to archetype isolates. Furthermore, each isolate contains multiple plasmids (Fig. S1), which are a similar size to previously described EPEC and ETEC virulence plasmids that carry the pathovar-specific virulence genes encoding BFP of EPEC^27, 33^, and LT or EatA of ETEC^19, 28, 30^.

### Phylogenomic analysis of the EPEC/ETEC isolates

To determine the genetic diversity of the EPEC/ETEC isolates we used whole-genome sequencing and phylogenomic analysis. The draft genome assemblies of the EPEC/ETEC hybrid isolates ranged in size from 5 to 5.37 Mb, with 144 to 255 contigs (Table 1). Identification of the seven multi-locus sequence typing (MLST) loci in each of the genomes demonstrated that the LT+ EPEC/ETEC isolates 102651, 102712, and 102771 are sequence type 328 (ST328), while the EatA+ EPEC/ETEC isolate 401140 is ST1788^34^ (Table 1). Thus, the LT+ EPEC/ETEC hybrid isolates are a different sequence type (ST) than the previously described LT+ EPEC/ETEC hybrid isolate 639^15^; however, they belonged to the same ST complex (ST278 complex), suggesting there is genomic similarity between the current EPEC/ETEC hybrid isolates and the previously described EPEC/ETEC hybrid isolate.

**Figure 1:**
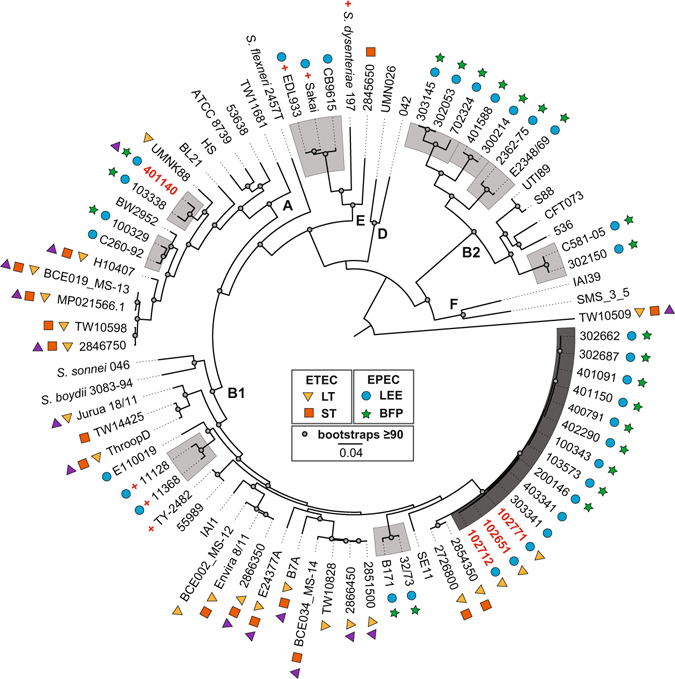
Phylogenomic analysis of the EPEC/ETEC hybrid isolates. The whole-genome sequences of the EPEC/ETEC hybrid isolates were compared with previously sequenced *E. coli* and *Shigella* genomes listed in Table S1 using a single nucleotide polymorphism (SNP)-based approach as previously described^17, 43^. SNPs were detected relative to the completed genome sequence of the laboratory isolate *E. coli* IAI39 using the *In Silico* Genotyper (ISG)^43^. A total of 159,709 conserved SNP sites, which were present in all of the genomes analyzed, were concatenated into a representative sequence for each genome. A maximum-likelihood phylogeny with 100 bootstrap replicates was inferred using RAxML v.7.2.8^56^. The presence of *E. coli* virulence genes in each of the genomes is indicated by symbols as follows: LT (yellow triangle), ST (orange square), LEE (blue circle), BFP (green star), EatA (purple triangle), and Shiga-toxin (red plus sign). The letters (A, B1, B2, D, E, and F) designate the *E. coli* and *Shigella* phylogroups that were previously defined^35, 36^. The EPEC/ETEC hybrid isolates are indicated in bold red. The phylogenomic lineages of the LEE-containing *E. coli* are indicated in light grey, while the EPEC7 lineage is in dark grey. Bootstrap values ≥90 are designated by a grey circle. The scale bar represents the distance of 0.05 nucleotide substitutions per site.

Phylogenomic analysis of the EPEC/ETEC isolates with 75 previously-sequenced *E. coli* and *Shigella* genomes (Table S1) demonstrated that the EPEC/ETEC hybrid isolates belonged to *E. coli* phylogroups A and B1^35, 36^ (Fig. 1, Table S1). Among the 75 reference genomes included in the phylogenomic analysis were 26 EPEC genomes representing eight of the previously described EPEC phylogenomic lineages^16, 18^. The reference genomes also included 23 genomically-diverse ETEC^21, 23, 28^, which formed distinct phylogenomic lineages from the EPEC (Fig. 1, Table S1). The three LT+ EPEC/ETEC hybrid isolates, 102651, 102712, and 102771, were part of the EPEC7 phylogenomic group, which also contained nine tEPEC (LEE+/BFP+) and two aEPEC (LEE+/BFP−) (Fig. 1, Table S1). The EatA+ EPEC/ETEC hybrid genome 401140 is part of the EPEC5 phylogenomic lineage within phylogroup A (Fig. 1). As demonstrated in previous comparative genomics studies^16, 18, 21, 23^, and further highlighted in this study, there is considerable genomic diversity among the *E. coli* isolates considered to be EPEC or ETEC based on their virulence gene and genomic content. This diversity now includes *E. coli* with virulence genes that are characteristic of both the EPEC and ETEC pathovars, emphasizing the dynamic nature of the virulence gene content of pathogenic *E. coli*, as well as the power of genomics to identify and characterize these emerging novel pathogens.

### Comparative genomics of the EPEC/ETEC isolates

To examine the extent of the genomic similarity of the EPEC/ETEC isolate genes relative to genes of traditional EPEC and ETEC isolates, we compared the genomic content of the EPEC/ETEC isolates with representative EPEC and ETEC genomes using large-scale BLAST score ratio (LS-BSR)(Table 2). There is the possibility for false-negative results using the LS-BSR method when genes are truncated at the end of contigs; however the impact on the comparisons is minimal and cannot be avoided using draft genome data. There were 1,617 genes that exhibited significant similarity (LS-BSR ≥ 0.8) in all of the 53 EPEC, ETEC, and EPEC/ETEC genomes analyzed (Table 2). This number is similar to the pangenome predictions for *E. coli* ^19, 22^, as well as the core genome size for diverse isolates by this comparative method^16, 18, 20, 21^. Meanwhile there were only four gene clusters that were identified in all of the EPEC genomes that were absent (LS-BSR < 0.4) from the ETEC genomes, and no gene clusters that were present in all ETEC and absent from the EPEC (Table 2, Table S2). There were 1,644 gene clusters in one or more of the EPEC genomes that were not in any of the ETEC genomes (Table 2). Interestingly, there were only 27 gene clusters that were unique to the three LT + EPEC/ETEC hybrid genomes that were not present in any of the other EPEC or ETEC genomes analyzed, including the most closely related genomes of the EPEC7 phylogenomic lineage (Table 2). These LT + EPEC7-specific genes included putative phage genes, type I restriction modification, and hypothetical proteins, suggesting these LT + EPEC/ETEC differed from the other EPEC7 by genes acquired via mobile elements (Table S2).

**Table 2:** LS-BSR analysis of the EPEC and ETEC isolates analyzed in this study

Group 1	Group 2	No. of Genomes (Group 1)	No. of Genomes (Group 2)	No. of LS-BSR Gene Clusters^a^		
				All Genomes^b^	≥50% of Genomes^b^	≥1 Genome^b^
EPEC^c^	ETEC	30	23	4	111	1,644
ETEC	EPEC^c^	23	30	0	17	2,499
ETEC + LT EPEC	other EPEC	26	27	0	19	2,607
EPEC7	other *E. coli*	14	39	1	13	190
LT EPEC	other *E. coli*	3	50	27	30	37

^a^The total number of core gene clusters (LS-BSR value ≥ 0.8) in all of the genomes (n = 53) analyzed was 1,617.

^b^The number of gene clusters that were present in all genomes, ≥50% of the genomes, or ≥1 of the genomes of Group 1 (LS-BSR ≥ 0.8) and absent from all of the genomes of Group 2 (LS-BSR < 0.4).

^c^The EPEC/ETEC isolates were included in the EPEC group due to their similarity to EPEC in the phylogenomic analysis.

*In silico* detection of *E. coli* virulence genes in the genomes of the EPEC/ETEC hybrid isolates compared with traditional EPEC and ETEC isolates demonstrated the genomes separated into pathovar-specific groups based on their virulence gene similarity (Fig. S2). The T3SS effectors and other EPEC virulence genes were primarily identified in the EPEC genomes, whereas the toxins and colonization factors were identified in the ETEC genomes (Fig. S2). Additional *E. coli* virulence genes that have been previously identified in multiple pathovars of *E. coli* such as the Type 2 and 6 secretion systems (T2SS, T6SS), as well as select autotransporters, were identified in the EPEC/ETEC hybrid genomes (Fig. S2). T2SS has been linked to virulence of EPEC in an animal model^37^, although its role in human disease is not well understood. In contrast, T2SS is a central component of LT+ ETEC, as it is required for the secretion of the LT toxin^38^, thus all LT+ ETEC isolates typically encode a T2SS (Fig. S2, Table S1). Additionally, all of the LT+ EPEC/ETEC genomes and other genomes of the EPEC7 phylogenomic lineage contained a T2SS, suggesting that the EPEC7 lineage contains the genomic content that would allow for the secretion of the LT toxin.

### *In silico* detection of plasmid genes in the EPEC/ETEC isolates

**Figure 2:**
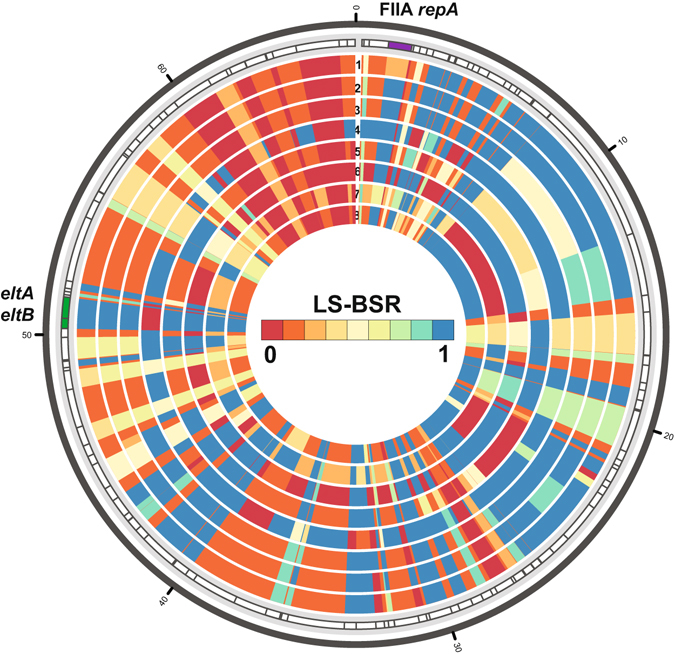
*In silico* detection of LT plasmid genes. The presence of protein-encoding genes with similarity to those of the previously sequenced LT-containing ETEC plasmid p666 from ETEC isolate H10407^28^, were identified in the EPEC/ETEC genomes using TBLASTN LS-BSR^17^. The outer track denotes the location of the protein-encoding genes of p666, while each of the inner tracks contains LS-BSR values indicating the presence (blue), divergence (yellow), or absence (red) of the genes in each of the genomes analyzed. The genomes analyzed are numbered as follows: 102651 (1), 102712 (2), 102771 (3), 402290 (4), E24377A (5), TW10509 (6), BCE019_MS13 (7), and B7A (8). The first four isolates are the EPEC/ETEC hybrid isolates and the other four isolates represent diverse ETEC isolates.

The plasmid content of *E. coli* often contains the virulence factors and thus the defining features of the pathovars, so the plasmid content of the EPEC/ETEC hybrids was examined using the completed plasmids from archetype ETEC^28^ and EPEC^17, 27^ isolates. The genes encoding the heat-labile toxin LT (*eltAB*), of the LT+ EPEC/ETEC isolates were nearly identical to those previously-characterized in the LT-encoding plasmid, p666, from the archetype ETEC isolate H10407^28^. *In silico* detection of genes with similarity to those of the LT-encoding plasmid, p666^28^, demonstrated that the *eltAB* genes of the LT+ EPEC/ETEC isolates are likely present on a plasmid with considerable genetic differences when compared to p666 (Fig. 2). This diversity of LT-encoding plasmids was also evident when comparing p666 to the phylogenomically-diverse LT encoding ETEC (E24377A, BEC019_MS13, TW10509, and B7A) (Fig. 2). Further clustering based on the LS-BSR values of the p666 plasmid genes in EPEC and ETEC genomes included in the phylogeny in Fig. 1 separated the isolates into three main groups (Fig. S3). Group I contained all but four of the LT+ ETEC isolates, as well as all of the LT+ EPEC/ETEC hybrid isolates (Fig. S3). Group II contained all but three of three of the EPEC isolates (Fig. S3). Finally Group III contained a mixture of isolates including three EPEC isolates, two ST+ ETEC isolates, and four LT+ ETEC isolates, including the two that are most similar to the EPEC7 lineage isolates (Fig. S3). The LT+ EPEC/ETEC hybrid isolates contained plasmids that are most similar to other ETEC in Group I (Fig. S3).

The other ETEC virulence factor, EatA, identified in EPEC/ETEC hybrid isolate 401140 has also been demonstrated to be encoded on a plasmid known as p948^28, 30^. Clustering analysis of the presence of the p948 genes demonstrated that the EPEC and ETEC isolates examined separated into five main groups (Fig. S4). ETEC isolates contained a greater proportion of p948 genes and are represented in groups I, III, and V, whereas groups II and IV contain the majority of the EPEC isolates, as well as the EPEC/ETEC hybrid isolate 401140 (Fig. S4). These findings demonstrate that there was greater genetic diversity observed among the EatA-encoding plasmids compared to the EatA + reference plasmid p948^28^ than observed for the LT-encoding plasmid (Figs S3 and S4).

One of the few genes on the ETEC virulence plasmids that was present in many of the EPEC and ETEC isolates was the replication protein-encoding gene, *repA* (Figs S5 and S6). The *repA* genes in these isolates are homologous to the *repA* in two plasmid groups, FIB and FIIA. Phylogenetic analysis of the identified FIB *repA* genes demonstrated these genes separated into two main groups (Fig. S5). Group A contained both EPEC and ETEC isolates, as well as the EPEC/ETEC hybrid isolates, whereas group B contained only EPEC isolates (Fig. S5). The LT+ EPEC/ETEC hybrid isolates FIB *repA* genes were most similar to *repA* genes of ETEC isolates, whereas, the FIB *repA* gene of the EatA+ EPEC/ETEC hybrid isolate 401140 grouped with *repA* genes from LEE+ /BFP+ EPEC isolates in group A (Fig. S5). It is possible that this FIB *repA* gene from 401140 belongs to the BFP-encoding plasmid of this isolate, since the FIB *repA* is associated with BFP-encoding plasmids^27, 33, 39^.

Phylogenetic analysis of all of the identified FIIA *repA* genes from genomes in the analysis with reference FIIA *repA* sequences from diverse *E. coli* isolates representing multiple pathovars demonstrated the pathovar-specificity of these *repA* sequences (Fig. S6). There were sub-groups consisting mostly of FIIA *repA* genes from EPEC isolates, and other sub-groups of genes from ETEC isolates (Fig. S6). However the LT+ EPEC/ETEC hybrid FIIA *repA* genes exhibit diversity, unlike their genomes and the FIB *repA* genes, which were similar (Figs S5 and S6). In contrast, the FIIA *repA* gene from the genome of the EatA+ EPEC/ETEC hybrid isolate 401140 was most related to the FIIA *repA* genes from nearly all of the same EPEC isolates that it was similar to in the FIB *repA* phylogeny (Figs S5 and S6). Overall, the plasmid content of the EPEC/ETEC hybrids suggests that the LT-carrying plasmids were likely acquired from an ETEC isolate.

### Comparative transcriptomics of the EPEC/ETEC hybrid isolates with archetype EPEC, ETEC, and commensal *E. coli* isolates

To investigate the global transcriptional responses of the EPEC/ETEC hybrid compared with EPEC and ETEC reference isolates, we performed RNA-Seq on these isolates during growth under laboratory conditions previously demonstrated to promote expression of *E. coli* virulence genes^40, 41^. The isolates examined included an EPEC reference isolate E2348/69, an reference EPEC7 isolate 402290, an ETEC reference isolate H10407, the human commensal isolate HS, and three of the EPEC/ETEC hybrid isolates (102651, 102712 and 401140) (Table S3). The EPEC/ETEC hybrid isolates and reference isolates were grown in high-nutrient (LB) and low-nutrient media (DMEM), as well as with and without added bile salts. We analyzed 52 RNA-Seq samples that generated over 3.2 billion reads (Table S3). We hypothesize that the EPEC/ETEC hybrid isolates will have global transcriptomes that most resemble that of phylogenomically-related EPEC isolates due to the similarity we observed for their genomic content.

**Figure 3:**
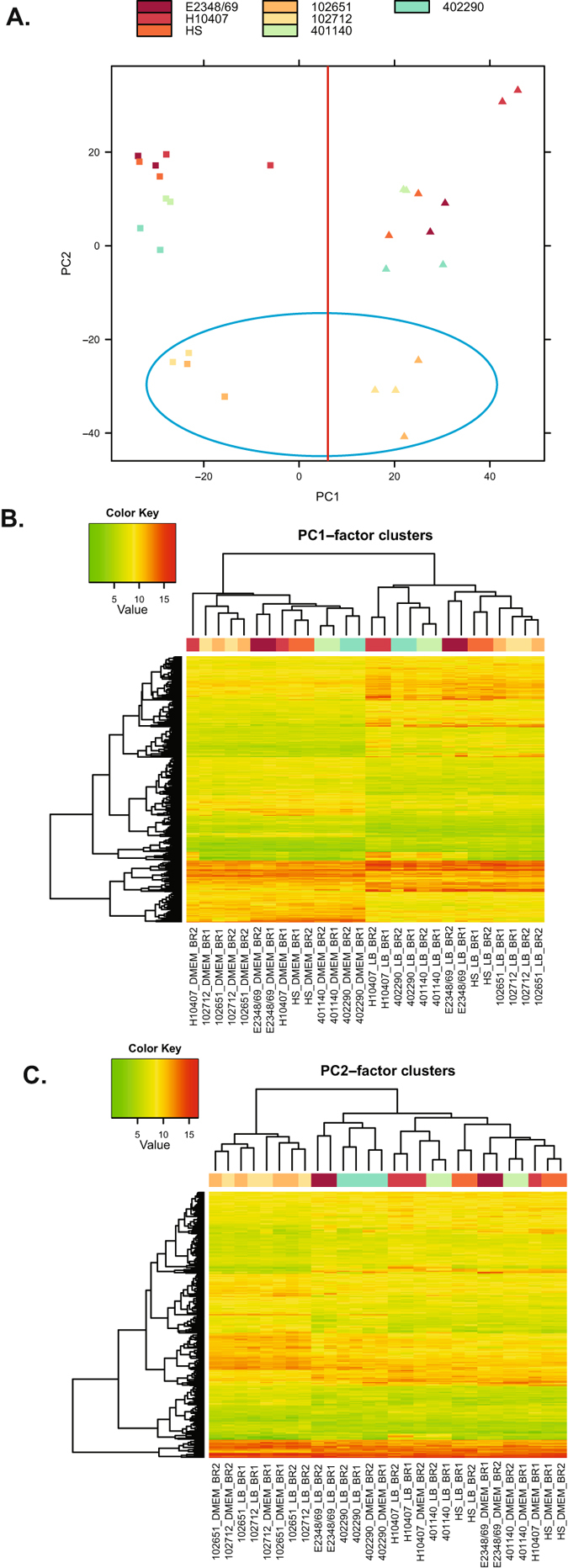
Analysis of the LB and DMEM RNA-Seq samples from all of the *E. coli* isolates. (**A**) Principal component (PC) analysis of the normalized expression values of the LB and DMEM samples for ETEC isolate H10407, non-pathogenic isolate HS, EPEC isolates E2348/69 and 402290, and the EPEC/ETEC hybrid isolates 102651, 102712, and 401140. Each of the RNA-Seq samples is indicated by a different color in the legend at the top of the panel. The squares represent the DMEM samples and the triangles represent the LB samples. The red line separates all of the LB and DMEM samples, and the blue circle identifies the samples of the LT+ EPEC/ETEC isolates 102651 and 102712. (**B)** Hierarchical clustering of 520 gene clusters of PC1 and (**C)** 263 gene clusters of PC2. The heatmaps in these two panels display the normalized gene expression values of the LS-BSR gene clusters that were present in all of the genomes and had the greatest deviation in their expression values.

Comparison of the RNA-Seq samples using principal component analysis demonstrated that they grouped by media type, with additional grouping of samples for the hybrid EPEC/ETEC isolates (Fig. 3, Fig. S7). The principal component (PC) scatter plot of the LB and DMEM samples without added bile salts for all isolates demonstrated there was greatest similarity among the samples by media type (Fig. 3A, divided by the red line, LB samples on the left and DMEM samples on the right) and grouping of the LT+ EPEC/ETEC hybrid isolates (Fig. 3A, blue circle). Clustering analysis of the 520 genes with the greatest standard deviation of expression in PC1 demonstrated a grouping by media type (Fig. 3B). Meanwhile clustering of the 263 genes with greatest deviation of expression in PC2 demonstrated similarity of the samples from the LT+ EPEC/ETEC hybrid isolates (102651 and 102712) (Fig. 3C). The PC scatter plot comparing samples of all growth conditions, including those with bile salts, also demonstrated the samples grouped by media (LB or DMEM) (Fig. S7A, red line). This grouping was highlighted by the clustering analysis of 333 genes with the greatest deviation of expression in PC1, which demonstrated that the samples grouped by media type (Fig. S7B). Clustering of 196 genes with the greatest deviation of expression in PC2 demonstrated similarity among the samples by isolate with grouping of samples for the LT+ EPEC/ETEC hybrid isolates 102651 and 102712 (Fig. S7C). These overall patterns of gene expression demonstrate that growth media has the greatest impact on the global transcriptional pattern, but the unique genomic content of each isolate is also a factor in the transcriptional outcomes.

From the global patterns of gene expression, the analysis was furthered to examine the transcriptional patterns for the isolate-specific genome content. The total number of genes that exhibited significant differential expression ranged from 10–519 depending on the isolate and growth condition (Table 3). The results of the comparative genomic analysis allowed us to define the core and accessory genomes included in the analysis. The number of differentially-expressed genes belonging to core gene clusters, which were present in all of the *E. coli* genomes analyzed using RNA-Seq, represented between 40–86% of the total number of differentially-expressed genes (Table 3). The number of isolate-specific genes, which were present in one genome (LS-BSR ≥ 0.8) and absent from the other genomes (LS-BSR < 0.4) ranged from 0–47 (Table 3). None of the differentially-expressed genes of the EPEC isolates or the EPEC/ETEC hybrid isolates belonged to EPEC-specific gene clusters, which were present in all of the EPEC (LS-BSR ≥ 0.8) and absent from the ETEC isolate H10407 and the non-pathogenic *E. coli* isolate HS (LS-BSR < 0.4) (Table 3). In contrast, there were 6–37 genes that exhibited significant differential expression for samples of the ETEC isolate H10407 that were present only in this isolate and were absent from the other *E. coli* including the EPEC/ETEC hybrid isolates (Table 3). There were up to six genes that had significant differential expression for gene clusters identified in ETEC isolate H10407 and the LT+ EPEC/ETEC isolates (Tables 3 and S4), including the *eltA* and *eltB*, which encode the alpha and beta subunits of LT, and had decreased expression (Table S4). There were 11 genes that were specific to genomes of the EPEC7 phylogenomic lineage isolates (402290, 102651, and 102712) that also exhibited significant differential expression in one or more of the isolates (Table 3). Among these EPEC7 genes that were expressed were genes encoding a putative lipoprotein and hypothetical proteins (Table S4). Also, there were eight differentially-expressed genes that were unique to the LT+ EPEC/ETEC isolates including a gene encoding a putative antitoxin, and Pap fimbrial proteins (Tables 3 and S4).

**Table 3:** Differential expression of conserved and unique genes in each of the *E. coli* isolates examined using RNA-Seq.

Isolate ID	Pathovar^a^	Treatments Compared^b^	LFC ≥ 2^c^	LFC ≤ −2^c^	Total DE Genes^c^	No. of DE Genes of Core Clusters^d^	No. of DE Genes of EPEC Clusters^e^	No. of DE Genes of ETEC Clusters^e^	No. of DE Genes of ETEC and LT + EPEC^e^	No. of DE Genes of EPEC7 Clusters^e^	No. of DE Genes of LT + EPEC Clusters^e^	No. of DE Genes of Isolate-Specific Clusters^f^
E2348/69	EPEC	DMEM vs. LB	180	251	431	270	0	NA	0	0	0	35
		DMEMB vs. LBB	178	156	334	207	0	NA	0	0	0	26
		DMEMB vs. DMEM	91	116	207	143	0	NA	0	0	0	18
		LBB vs. LB	101	175	276	212	0	NA	0	0	0	16
H10407	ETEC	DMEM vs. LB	211	308	519	379	NA	23	4	0	0	23
		DMEMB vs. LBB	50	152	202	120	NA	32	3	0	0	32
		DMEMB vs. DMEM	21	77	98	28	NA	37	1	0	0	37
		LBB vs. LB	82	61	143	105	NA	6	0	0	0	6
HS	Non-pathogenic	DMEM vs. LB	248	250	498	416	0	NA	0	0	0	7
		DMEMB vs. LBB	166	305	471	407	0	NA	0	0	0	6
		DMEMB vs. DMEM	49	121	170	140	0	NA	0	0	0	14
		LBB vs. LB	163	101	264	209	0	NA	0	0	0	5
402290	EPEC	DMEM vs. LB	238	268	506	378	0	NA	0	4	0	4
		DMEMB vs. LBB	NA	NA	NA	NA	NA	NA	NA	NA	NA	NA
		DMEMB vs. DMEM	NA	NA	NA	NA	NA	NA	NA	NA	NA	NA
		LBB vs. LB	NA	NA	NA	NA	NA	NA	NA	NA	NA	NA
102651	EPEC/ETEC	DMEM vs. LB	86	203	289	216	0	NA	4	7	5	0
		DMEMB vs. LBB	32	94	126	95	0	NA	0	2	1	0
		DMEMB vs. DMEM	25	13	38	18	0	NA	0	4	4	0
		LBB vs. LB	9	7	16	10	0	NA	0	0	3	0
102712	EPEC/ETEC	DMEM vs. LB	122	163	285	202	0	NA	1	4	2	2
		DMEMB vs. LBB	114	261	375	249	0	NA	6	11	8	2
		DMEMB vs. DMEM	45	119	164	79	0	NA	1	3	4	3
		LBB vs. LB	8	2	10	4	0	NA	0	0	4	0
401140	EPEC/ETEC	DMEM vs. LB	145	189	334	266	0	NA	0	0	0	11
		DMEMB vs. LBB	151	195	346	272	0	NA	0	0	0	12
		DMEMB vs. DMEM	331	123	454	267	0	NA	0	0	0	47
		LBB vs. LB	224	67	291	146	0	NA	0	0	0	36

^a^The pathovar designation based on virulence gene content.

^b^The RNA-Seq samples compared by DESeq analysis.

^c^LFC is the Log_2_ fold-change of the genes that exhibited significant (LFC ≥ 2 or ≤−2 and FDR ≤ 0.05) differential expression (DE).

^d^The total number of core gene clusters (LS-BSR ≥ 0.8 in all genomes analyzed by RNA-Seq) is 3,559.

^e^The number of DE genes that were identified by LS-BSR analysis as present (LS-BSR ≥ 0.8) in all genomes of the described group and absent (LS-BSR < 0.4) from all other genomes.

^f^The isolate-specific genes are those that were in one genome with an LS-BSR value ≥0.8 and <0.4 in the other genomes.

**Figure 4:**
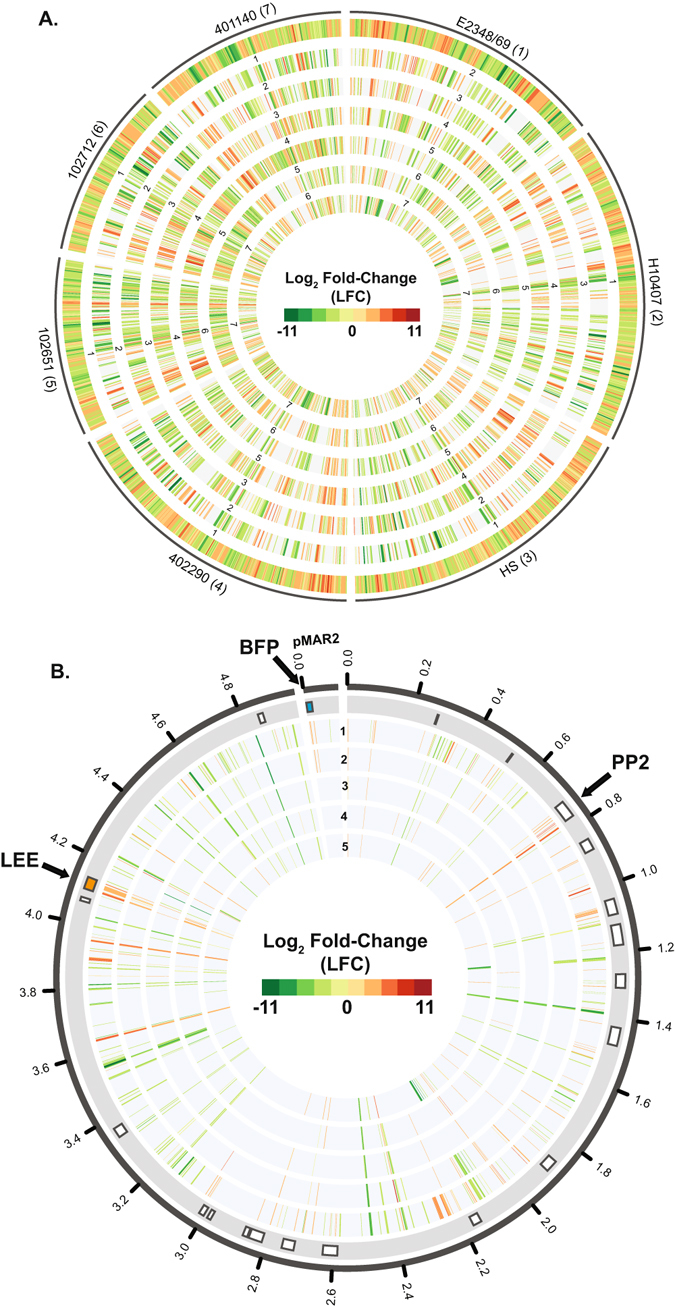
Comparative transcriptomics of the EPEC/ETEC hybrid isolates with representative EPEC and ETEC isolates. (**A**) Circos plot comparing the global transcriptomes of the EPEC/ETEC isolates (102651, 102712, and 401140) with representative *E. coli* isolates (H10407 (ETEC), HS (commensal), E2348/69 (EPEC1), and 402290 (EPEC7)) during growth in DMEM compared to LB. The outermost track contains all of the genes that exhibited significant differential expression for each of the indicated nine EPEC isolates. The inner tracks contain the LFC values of the same LS-BSR gene cluster as the gene in the outermost reference track, and the number of each of the inner tracks indicates the *E. coli* isolate designated in the outer track. The genes that were not present in the other EPEC isolates, or did not exhibit significant differential expression are absent from the inner tracks. (**B**) Circos plot comparing the global transcriptomes of only the EPEC isolates. The RNA-Seq data tracks are numbered as follows: E2348/69 (1), 402290 (2), 102651 (3), 102712 (4), and 401140 (5). The boxes in the outermost track indicate the location of insertion element and prophage (PP) regions in the chromosome of E2348/69^27^. The LEE region (orange) of the chromosome and BFP of the plasmid, pMAR2 (blue) are indicated by arrows. Data tracks 2–5 contain the LFC values of the same LS-BSR gene cluster as the genes of E2348/69 (track 1) that exhibited significant differential expression. The genes that were not identified in the other EPEC isolates, or did not exhibit significant differential expression are absent from tracks 2–5.

Comparison of the global transcriptomes of the EPEC/ETEC hybrid isolates with the reference EPEC, ETEC, and a non-pathogenic *E. coli* isolate HS in DMEM compared to LB demonstrated that overall there were similar transcriptional patterns among all the *E. coli* isolates, but also many examples of isolate-specific transcriptional responses (Fig. 4A). Further comparison of all differentially-expressed genes of EPEC isolates belonging to the same LS-BSR gene clusters as the differentially-expressed genes of the EPEC reference isolate E2348/69 demonstrated similar patterns of increased or decreased expression among these conserved EPEC genes (Fig. 4B). Some genes exhibited the same trend of increased or decreased expression in all of the EPEC, as well as in the EPEC/ETEC hybrid isolates (Fig. 4B). For instance, genes of the LEE and prophage 2 (PP2) regions exhibited increased expression in all of the EPEC and hybrid isolates (Fig. 4B). This comparison of only the EPEC isolates demonstrates there is consistency among the global transcriptomes of the isolates with an EPEC genomic backbone (Fig. 4B); however, this comparison only includes the highly conserved genes. In contrast, the comparison that included ETEC and the non-pathogenic isolate HS, demonstrated there was differential expression of additional shared genes in DMEM compared to LB for the EPEC/ETEC hybrid isolates (Fig. 4A). This is a trend that has been observed previously^17, 42, 43^. Thus, the RNA-Seq analyses revealed that the global transcriptomes of the EPEC/ETEC hybrid isolates are most similar to the phylogenomically-related EPEC7 isolate.

### Functional characterization of known EPEC and ETEC virulence genes

**Figure 5:**
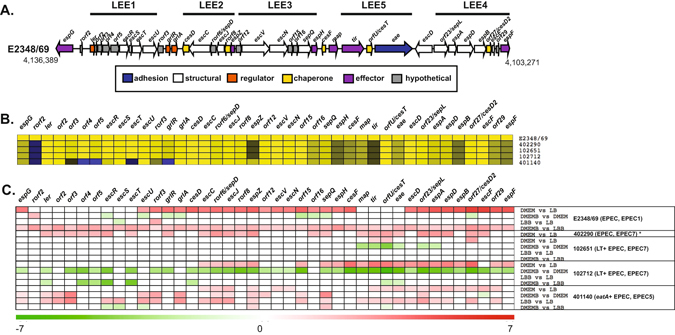
Differential expression of protein-coding genes within the LEE region. (**A**) Diagram of the protein-encoding genes within the LEE region of EPEC isolate E2348/69. The predicted protein function is indicated by the color of the arrow, and the size and direction of the arrows indicates the size of each predicted gene and the direction of transcription. (**B**) *In silico* detection of the protein-encoding genes of the LEE region of EPEC isolate E2348/69 in each of the EPEC genomes analyzed in this study. The colors of the heatmap represent the BSR values of each gene, with yellow indicating a gene is present, blue indicating a gene is absent, and grey to black indicating sequence divergence. (**C**) Heatmap of the differential expression for each of the sample comparisons of the EPEC isolates (E2348/69 and 402290) and the EPEC/ETEC hybrid isolates (102651, 102712, and 401140). Only significant LFC values ≥1 or ≤−1 are represented in the heatmap. Red indicates increased differential expression while green indicates decreased differential expression. White indicates that a gene was either not present in the EPEC isolate, and/or did not exhibit significant differential expression. *The differential expression of EPEC isolate 402290 was previously investigated during growth in DMEM vs. LB (Hazen *et al*. unpublished) and was included as a reference comparison in the current study; however, this EPEC isolate was not grown in LB and DMEM with added bile.

Differences in the global transcriptional responses of the EPEC/ETEC hybrid isolates compared with reference EPEC and ETEC isolates were further investigated by comparing known EPEC and ETEC virulence genes. It was anticipated that the virulence gene expression of the EPEC/ETEC hybrids would be similar to the expression in the reference EPEC and ETEC isolates. All of the LEE genes previously described for E2348/69 (Fig. 5A) were identified in the genomes of the EPEC/ETEC hybrid isolates 102651 and 102712 with the exception of *rorf2*, which was absent in the final annotation and thus in the by LS-BSR analysis (Fig. 5B). Meanwhile, the EPEC/ETEC hybrid isolate 401140 from the EPEC5 phylogenomic lineage was lacking several genes of the LEE including *grlR* (Fig. 5B). In each case, reads that map to each of the genes were present, but the genes were lacking in the final annotation and thus not included in the final analysis.

The majority of the LEE genes in the EPEC/ETEC hybrid isolates had increased expression in DMEM compared to LB, similar to that observed for the LEE+ /BFP+ EPEC reference isolates E2348/69 and 402290 (Fig. 5C). Interestingly, LEE genes of the reference EPEC isolate E2348/69, as well as the EPEC/ETEC hybrid isolates 102651 and 102712 exhibited decreased expression following growth in LB or DMEM with bile salts when compared to growth in the same media without added bile salts (Fig. 5C). In contrast, all but one of the LEE genes that had significant differential expression in the EPEC/ETEC hybrid isolate 401140, exhibited increased expression during growth in LB or DMEM with bile salts (Fig. 5C).

We also investigated the differential expression of protein-encoding genes within the BFP region in EPEC/ETEC hybrid isolate 401140, which was the only EPEC/ETEC hybrid isolate to contain the BFP genes (Fig. S8). The genome of EPEC/ETEC hybrid isolate 401140 contained the BFP region of reference plasmid pMAR2 from EPEC isolate E2348/69^27^(Fig. S8A and B), as well as the plasmid-encoded regulator *perC;* however, it lacked the *perA* and *perB* regulatory genes^44, 45^ (Fig. S8B). The BFP genes of EPEC isolate E2348/69 exhibited increased differential expression in DMEM compared to LB, both with and without bile (Fig. S8C). This is consistent with previous reports that BFP genes have increased expression during growth in nutrient-limited media (DMEM) compared to growth in nutrient-rich media in EPEC isolates (LB)^40, 41, 46^. Similar to that observed for the LEE genes of E2348/69, two BFP genes, *bfpI* and *bfpK*, had decreased expression during growth in LB with bile compared to growth in LB without bile (Fig. S8C). In contrast, the BFP genes of EPEC/ETEC hybrid isolate 401140 had decreased expression during growth in DMEM compared to LB with and without bile (Fig. S8C). Also, opposite to what was observed for EPEC isolate E2348/69, the BFP genes of EPEC/ETEC hybrid isolate 401140 had increased expression in DMEM with bile or LB with bile compared to the same media type without bile (Fig. S8C).

**Figure 6:**
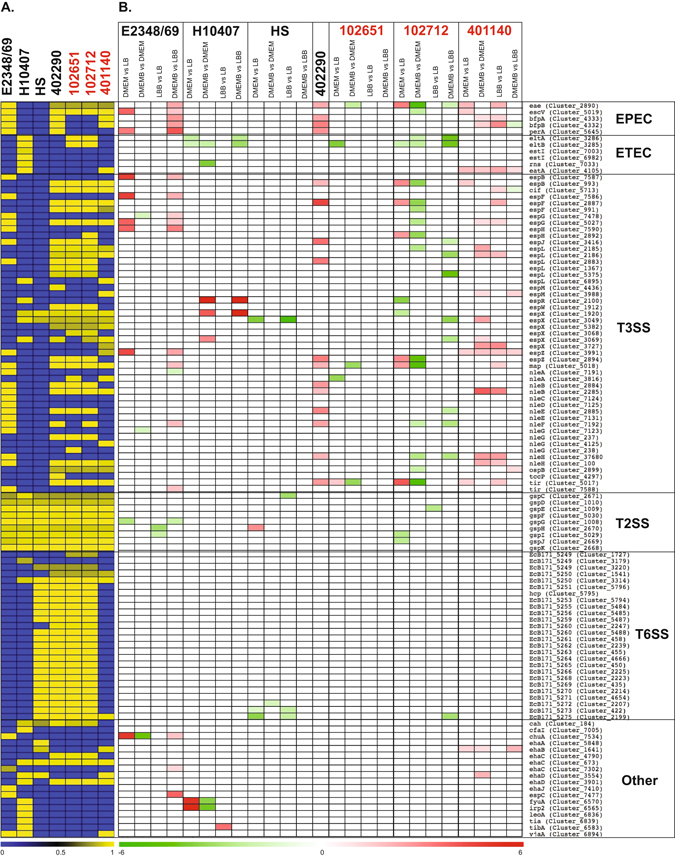
Differential expression of *E. coli* virulence genes. (**A**) *In silico* detection of *E. coli* virulence genes in each of the *E. coli* genomes analyzed in this study using RNA-Seq. The colors of the heatmap represent the BSR values of each gene with yellow indicating a gene is present and blue indicating a gene is absent. (**B**) Heatmap of the differential expression of the virulence genes for each of the sample comparisons of the *E. coli* isolates. Only significant log_2_ fold-change (LFC) values ≥1 or ≤−1 are represented in the heatmap. Red indicates increased differential expression while green indicates decreased differential expression. White indicates that a gene was either not present in the *E. coli* isolate, and/or did not exhibit significant differential expression. The only sample comparison of EPEC isolate 402290 that is shown is for DMEM compared to LB since this isolate was analyzed previously and was not grown with added bile salts (Hazen *et al*. unpublished).

To determine whether additional virulence-associated genes are present and expressed in the EPEC/ETEC hybrid isolates we used LS-BSR to identify previously characterized *E. coli* virulence genes in the genomes of the hybrid isolates (Fig. S2, Fig. 6A), and determined whether these genes had significant differential expression under the growth conditions tested (Fig. 6B). The genomes of the three EPEC/ETEC hybrid isolates (102651, 102712, and 102771) all contained the additional non-LEE-encoded effectors including EspL, NleB, and NleH (Fig. S2, Fig. 6A). As described above, the LEE genes exhibited increased expression in the EPEC/ETEC hybrid isolates following growth in DMEM compared to LB and decreased expression when grown in the presence of bile compared to without bile (Fig. 5C), and the trend extends to the Nle genes.

The only ETEC virulence-associated genes identified in the genomes of the EPEC/ETEC hybrid isolates were *eltA* and *eltB* encoding LT, and the gene encoding the autotransporter EatA (Fig. 6A). The general secretion system (T2SS) is also required for secretion of the LT^38^, and genes encoding a T2SS were present in the genomes of the LT+ EPEC/ETEC hybrid isolates (Fig. S2, Fig. 6A). Although there was increased expression of the EPEC LEE genes following growth in DMEM compared to LB, the LT-encoding genes, *eltA* and *eltB*, had decreased expression in under these conditions, as well as growth in the media with and without bile depending on the isolate and growth media (Fig. 6B). These results suggest that the LEE and LT, the major virulence factors of EPEC and ETEC, respectively may not be regulated under the same growth conditions in the laboratory.

Another class of virulence gene present and expressed in the EPEC/ETEC hybrid isolates were autotransporters (Fig. S2, Fig. 6A). Among the autotransporters identified were the ETEC autotransporter EatA, and several autotransporters that are typically found in EHEC (*ehaB, ehaC*, and *ehaD*) (Fig. S2, Fig. 6A). The EatA-encoding gene homolog was identified only in EPEC/ETEC hybrid isolate 401140 (Fig. S2, Fig. 6A). Meanwhile at least one homolog of the EhaB, EhaC, and EhaD-encoding genes were present in each of the four of the EPEC/ETEC hybrid isolates (Fig. S2, Fig. 6A). These *eha* genes were originally characterized in EHEC isolates^47^, but have now been identified in diverse *E. coli* isolates^48^. Although these autotransporters were identified in all of the EPEC/ETEC hybrid isolates they only exhibited significant differential expression in the EPEC/ETEC hybrid isolate 401140 (Fig. 6B). These findings demonstrate the possibility of the simultaneous expression of EPEC (LEE or BFP) and ETEC (EatA) virulence genes in an *E. coli* clinical isolate that is most genomically-related to traditional EPEC isolates.

### Functional characterization of the EPEC and ETEC virulence genes

**Figure 7:**
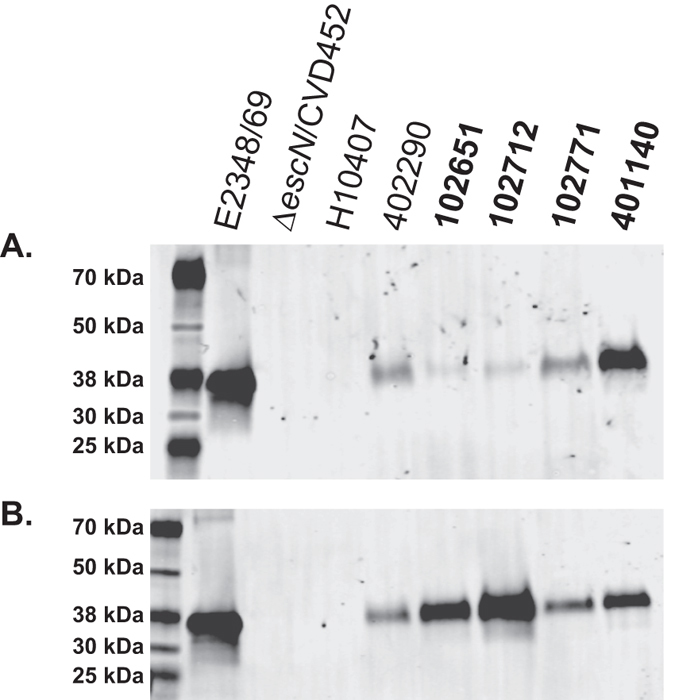
Functional characterization of T3SS in the EPEC/ETEC hybrid isolates. Immunoblot analysis for EspB in the supernatants of traditional EPEC (E2348/69 and 402290), ETEC (H10407), and the EPEC/ETEC hybrid isolates (102651, 102712, 102771, and 401140) grown in DMEM with high glucose (**A**) or with low glucose (**B**). Included as a negative control is *E. coli* CVD452, which is an isogenic T3SS-deficient ∆*escN* mutant of E2348/69^49^ and ETEC (H10407) which lacks a T3SS^28^. The first lane of each image contains the Chameleon Duo pre-stained protein ladder (Li-Cor). Under both conditions all of the EPEC/ETEC isolates have a functional T3SS as determined by the EspB secreted protein. The EPEC/ETEC isolates are indicated in bold.

**Figure 8:**
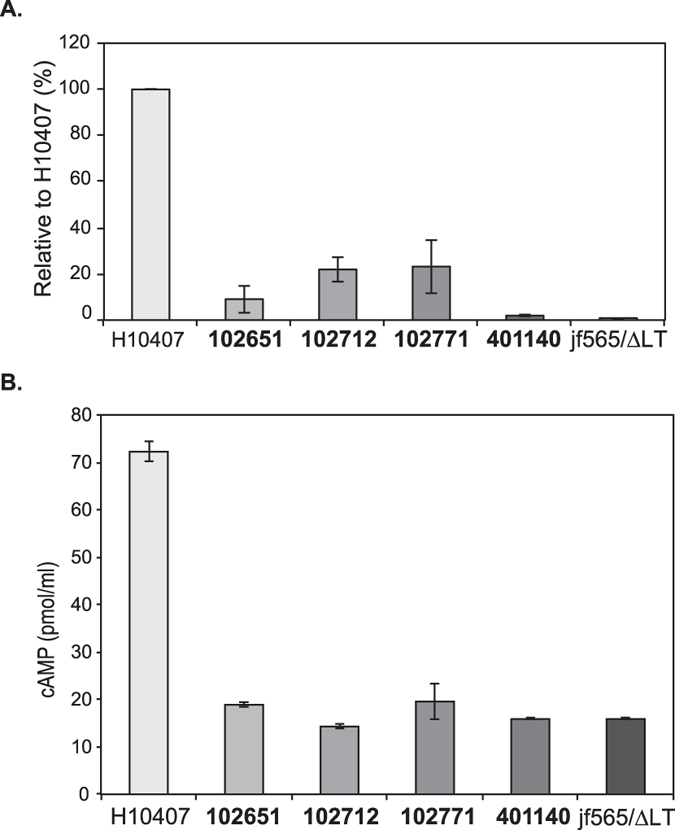
ETEC LT secretion and cAMP activation in target epithelial cells. (**A**) LT in culture supernatants from the EPEC/ETEC hybrids compared to the prototype ETEC isolate H10407, as measured by a GM1-ganglioside binding assay. The negative controls are isolate 401140 and jf565/ΔLT, which lack the *eltAB* genes. (**B**) Functional effectiveness of heat-labile toxin was determined by examination of cAMP production in target Caco-2 epithelial cells following infection with mutant strains relative to the wild type H10407, or the jf565 *eltAB* mutant. The EPEC/ETEC isolates are indicated in bold.

To investigate whether the EPEC and ETEC virulence genes are functional in the EPEC/ETEC hybrid isolates, we assayed for the secretion of the EPEC T3SS effector, EspB (Fig. 7), and the ETEC LT toxin (Fig. 8). These assays would provide evidence that the virulence factors that are present in the genome and expressed in the transcriptome studies, are also being produced by the EPEC/ETEC isolates. We hypothesize that the EPEC/ETEC isolates have the potential to produce the canonical virulence factors from both pathovars. Immunoblot analysis for secretion of EspB by the T3SS was performed on culture supernatants following growth to an OD_600_ of ~1.0 in DMEM supplemented with either high glucose (Fig. 7A), or with low glucose (Fig. 7B). Immunoblotting revealed EspB was secreted into the bacterial supernants of all samples examined, with the exception of the negative controls: CVD452, an isogenic T3SS-deficient ∆*escN* mutant of E2348/69^49^, and ETEC isolate H10407, which does not encode a T3SS (Table S1)^28^. There appeared to be greater secretion of EspB following growth in DMEM with low glucose when compared to growth in DMEM with high glucose (Fig. 7). These results are congruent with the RNA-Seq results, which demonstrate the T3SS genes of the LEE have increased expression in the DMEM media (Figs 5–6). These findings demonstrate that the T3SS of the EPEC/ETEC hybrid isolates is functional and can both produce and secrete EPEC T3SS effectors.

To determine whether the LT+ EPEC/ETEC hybrid isolates could produce and secrete the LT toxin, we used an ELISA to detect the presence of the holotoxin subunit (EltA) protein in the supernatant during laboratory growth (Fig. 8A). The EltA protein was detected, although at low levels, in the LT+ EPEC/ETEC hybrid isolates compared to ETEC isolate H10407 (Fig. 8A). However, the secreted LT toxin of the EPEC/ETEC hybrid isolates did not exhibit significant cAMP activity (Fig. 8B). The presence of a functional T2SS, which is required for secretion of LT and YghJ, was verified in the LT+ EPEC/ETEC hybrid isolates (Fig. S9A). The LT inactivity is likely due to the non-synonymous mutation present in the *eltA* holotoxin subunit resulting in the introduction of a premature stop codon in the *eltA* coding region (Fig. S10). Thus, we were able to confirm that at least a portion of the *eltA* gene is transcribed and translated but the potentially truncated EltA subunit results in the secretion of an enzymatically inactive holotoxin by the EPEC/ETEC hybrid isolates.

Immunoblot analysis of EatA indicated this protein was not produced by the EatA+ EPEC/ETEC hybrid isolate 401140 during growth in CAYE medium (Fig. S9B), which has been used in studies investigating ETEC virulence factors^50, 51^. In contrast, the *eatA* gene had increased expression during growth in DMEM along with the EPEC virulence genes. This finding demonstrates that the EatA protein-encoding gene can be acquired and regulated by native EPEC transcriptional regulators. Further investigation is necessary to determine whether certain transcriptional regulators may be simultaneously regulating both EPEC and ETEC virulence genes.

Overall, the findings from the functional analyses demonstrate that the canonical virulence factors from multiple pathovars can be maintained, expressed, and secreted by a single bacterial isolate.

## Discussion

The identification of hybrid isolates carrying virulence factors from multiple pathovars of *E. coli* is becoming more common^14, 15^, as investigators screen large collections for larger panels of diverse virulence factors. The most impactful hybrid isolate to be identified to date is from the European O104:H4 outbreak in 2011^7, 12, 13^. Using a combination of comparative genomics, transcriptomics, and functional characterization of virulence mechanisms, we demonstrate that these four EPEC/ETEC hybrid isolates are likely EPEC isolates that have acquired ETEC virulence genes via mobile genetic elements, most likely divergent plasmids. The four EPEC/ETEC hybrid isolates all contain chromosomally-encoded virulence genes of EPEC such as intimin and T3SS of the LEE, and other non-LEE encoded effectors (Table S1, Fig. S2)^3, 6, 27, 49^. The EPEC/ETEC hybrid isolates also contain plasmid-encoded ETEC virulence genes that encode LT and EatA (Table S1, Fig. S2)^30–32^. Although these isolates contain a T2SS required for secretion of the LT toxin, which is typically encoded on the chromosome of ETEC. The EPEC/ETEC hybrid isolates lack other ETEC virulence genes, such as traditional colonization factor antigens. Phylogenomic analysis further highlighted the greater genomic similarity of the hybrid isolates to traditional EPEC clinical isolates versus the ETEC isolates. These findings demonstrate that the EPEC/ETEC hybrid isolates are likely EPEC that have horizontally-acquired some ETEC virulence genes. However a larger study will be required to determine the stability and advantages of maintaining the canonical virulence factors of multiple pathovars in the same genetic background.

The findings from the plasmid analyses highlight the considerable genetic diversity of the ETEC virulence plasmids present in the diverse ETEC genomes as well as the EPEC/ETEC hybrid genomes. This is consistent with the findings of previous studies that have characterized the sequences of ETEC plasmids^19, 28, 52^. Although some genes of the LT-encoding plasmid p666 were absent from or exhibited sequence divergence in the LT+ EPEC/ETEC genomes, the p666 genes identified in the LT+ EPEC/ETEC hybrid genomes were most related to plasmid genes of other ETEC rather than of EPEC or other *E. coli* pathovars. Taken with the nearly identical nucleotide sequence of the LT-encoding genes of the EPEC/ETEC hybrid isolates to the LT-encoding genes of previously described ETEC, these findings suggest the EPEC/ETEC hybrid isolates have likely acquired an LT-encoding plasmid.

To verify that the EPEC and ETEC virulence genes identified in the EPEC/ETEC hybrid isolates have the potential to contribute to the pathogenesis of these isolates we used RNA-Seq and secretion assays to investigate their functionality. The RNA-Seq and comparative transcriptomics demonstrated that the EPEC/ETEC hybrid isolates are able to express the virulence genes of both EPEC and ETEC. Furthermore, the EPEC-specific T3SS was verified to be functional in these isolates by the secretion of the EspB effector. Although these isolates contain the virulence genes necessary for production and secretion of LT, the *eltA* genes of these hybrid isolates contain a non-synonymous change that appears to result in the secretion of a truncated gene product, which impairs the enzymatic function of LT.

In summary, the combined approach of using comparative genomics, transcriptomics, and functional characterization of virulence genes demonstrated the virulence potential of these four EPEC/ETEC hybrid isolates. These EPEC/ETEC hybrid isolates may represent an opportunistic *E. coli* pathogen that can occupy the pathovar-specific disease niche of either EPEC, which causes disease primarily in infants and young children, or that of ETEC, which causes disease in people of all ages^4, 6, 7, 53^. Alternately, these EPEC/ETEC hybrid isolates may represent a chance acquisition of an LT-containing plasmid by EPEC, and the plasmid and/or LT-encoding genes may be transiently maintained, or genetically inactivated. These findings highlight the occurrence of *E. coli* pathovar hybrids that may be overlooked during clinical characterization or research studies that are looking for the presence of the canonical virulence genes belonging to a single pathovar. This study also further demonstrates the utility of whole-genome sequencing and phylogenomic analysis for characterizing the *E. coli* pathovar hybrids. We would anticipate that as sequencing becomes more routinely used in clinical diagnostics, the identification of unanticipated combinations of virulence factors that have previously been considered to be exclusive in one pathovar or another will occur more frequently.

## Materials and Methods

### Bacterial isolates and media

The LT+ (102712, 102771, and 102651) and EatA+ (401140) EPEC/ETEC hybrid isolates examined in this study were isolated through the Global Enteric Multisite Study (GEMS)^25^. The EPEC/ETEC isolates were grown in Lysogeny Broth (LB)^54^ (Difco), or in Dulbecco’s Modified Eagle’s Medium (DMEM) supplemented with high glucose (4.5 g/L) or low glucose (1 g/L)(Invitrogen). Bile salts were supplemented at 3% (wt/vol) in described media.

### Genome sequences

The genomes of the three LT+ EPEC/ETEC isolates (102651, 102712, and 102771) analyzed in this study were sequenced as previously described^16^. The genome of the EatA+ EPEC/ETEC isolate (401140) was generated in a previous study^16^. All genome accession numbers are listed in Table S1.

### Multilocus sequence typing

The seven loci (*adk, gyrB, fumC, icd, mdh, purA*, and *recA*) of the multilocus sequence typing (MLST) scheme developed by Wirth *et al*.^34^ were located in the genomes of each of the EPEC/ETEC hybrid isolates. These loci were compared with the database maintained by the University of Warwick (http://mlst.warwick.ac.uk/mlst/dbs/Ecoli) to obtain the sequence type of each of the hybrid isolates.

### Phylogenomic analysis

The genomes of the four EPEC/ETEC isolates analyzed in this study were compared with 75 previously sequenced *E. coli* and *Shigella* genomes (Table S1) using the *In Silico* Genotyper (ISG)^17, 43^. Single nucleotide polymorphisms (SNPs) were detected relative to the completed genome sequence of the phylogroup F laboratory isolate *E. coli* IAI39 (NC_011750.1) using the *In Silico* Genotyper (ISG)^43^, which uses NUCmer v.3.22^55^ for SNP detection. The SNP sites that were identified in all genomes analyzed were concatenated and used to construct a maximum-likelihood phylogeny using RAxML v7.2.8^56^. The phylogeny was constructed using the GTR model of nucleotide substitution with the GAMMA model of rate heterogeneity, and 100 bootstrap replicates. The phylogeny was then visualized using FigTree v1.4.2 (http://tree.bio.ed.ac.uk/software/figtree/).

### Large Scale-BLAST Score Ratio (LS-BSR) analysis

The genomes of the 53 EPEC and ETEC isolates including the four EPEC/ETEC hybrid isolates (Table S1) were compared using LS-BSR as previously described^16, 57^. The predicted protein-encoding genes of each genome that had ≥80% nucleotide identity to each other were assigned to gene clusters using uclust^58^. Representative sequences of each gene cluster were then compared to each genome using TBLASTN^59^ with composition-based adjustment turned off, and the TBLASTN scores were used to generate a BSR value indicating the detection of each gene cluster in each of the genomes (Table S1). The BSR value was determined by dividing the score of a gene compared to a genome by the score of the gene compared to its own sequence. The LS-BSR values and the nucleotide sequences of each gene cluster for the 53 EPEC and ETEC isolates are included in Supplemental Data Sets S8 and S9.

### Plasmid analyses

The four EPEC/ETEC isolates were examined for their plasmid content using an acid-phenol extraction method as previously described^17^. The extracted plasmid DNA was run on a 0.7% w/v agarose gel for four hours and was visualized following staining and de-staining with ethidium bromide. The plasmid content of the EPEC/ETEC isolates was compared with reference strains of EPEC (E2348/69) and ETEC (H10407 and E24377A).

Plasmid genes of the previously sequenced ETEC plasmids p666 and p948 from reference strain H10407 were detected by *in silico* analysis in each of the EPEC/ETEC isolates using LS-BSR as described above. Heatmaps illustrating the presence or absence of the plasmid genes were generated using MeV.

### Gene alignments and phylogenies

Individual genes identified in the genomes of the EPEC/ETEC hybrid isolates including the FIB *repA*, FIIA *repA*, and *eltA* genes, were compared to those of previously described EPEC and/or ETEC isolates by alignment and phylogenetic analysis as previously described^17^. The nucleotide sequences were aligned by ClustalW of MEGA6^60^. A maximum-likelihood phylogeny was constructed using the Kimura 2-parameter model and 1,000 bootstraps.

### RNA isolation and sequencing

The EPEC isolates were grown overnight in LB and were inoculated 1:100 into 50 ml of LB, or DMEM supplemented with 4.5 g/L glucose and 3% (wt/vol) bile salts, in a 250 ml flask. The flasks were grown at 37 °C with shaking (225 rpm) to a final optical density (OD_600_) of approximately 0.5, corresponding to the exponential growth phase. The cells were pelleted from a total of 10 ml of the culture medium by centrifuging at 3,500 × g for 5 min, and the supernatant was discarded. Total RNA was isolated from the cell pellet using the Ribopure bacteria kit (Ambion) and treated with the Ribopure DNase I to remove contaminating DNA. The samples were then treated with the Turbo DNA-free kit (Ambion) to ensure all contaminating DNA was removed. RNA samples were verified to be DNA free by qPCR analysis for the conserved *rpoA* gene as previously described^42^. The DNA-free RNA samples were used for library construction with the Ovation Prokaryotic RNA-Seq System (NuGen), and sequenced using 100 bp paired-end sequencing on the Illumina HiSeq.

### RNA-Seq analyses

The Illumina reads generated for each RNA sample were analyzed and compared using an in-house Ergatis-based^61^ RNA-Seq analysis pipeline as previously described^42^. The completed genome sequence and annotation that is publicly available for EPEC isolate E2348/69 was used for the RNA-Seq analysis of this isolate. The draft genome assemblies of 102651, 102712, and 102771 were annotated using an in-house Ergatis-based^61^ annotation pipeline^62^. The RNA-Seq reads from each biological sample were aligned to their respective genome sequences using Bowtie^63^, and the number of reads that aligned to the protein-encoding regions and intergenic regions was determined using HTSeq^64^. The differential expression of each gene in two different treatments (DMEM vs. LB) was then determined using DESeq^65^ by adjusting for differences in the library sizes between samples, averaging across biological replicates, and calculating the log_2_ fold-change (LFC) values and their corresponding p-values with false discovery rate-based correction. The genes were then filtered for further analysis by meeting the following criteria: LFC ≥ 2, ≤−2, a minimum read count percentage, and false discovery rate (FDR) ≤ 0.05. Genes that met these criteria were identified as having significant differential expression during growth in DMEM compared to LB.

The protein-encoding regions of the seven *E. coli* isolates analyzed using RNA-Seq (Table S3) were compared using LS-BSR as described above. The LS-BSR values and the nucleotide sequences of each gene cluster for the seven *E. coli* only are included in Supplemental Data Sets 10 and 11.

The circular displays of the significant LFC values were generated using Circos 0.67–6^66^. The outermost track contains the differential expression values (LFC) for all genes that exhibited significant differential expression for each of the *E. coli* isolates, while the inner tracks contain the expression values of genes that belonged to the same gene cluster by LS-BSR analysis as the corresponding gene in the outer track. Heatmaps of the significant LFC values for the LEE and BFP genes were generated using MeV^67^.

The LS-BSR gene clusters of the *E. coli* isolates were used to examine difference in gene expression by principal component analysis and hierarchical cluster analysis. The analysis was performed using in-house Perl scripts and heatmaps were generated using R statistical package v2.15.2 that in turn used the DESeq v1.10.1^68^ library for normalization and the gplots v2.11.0 library for generating the heat maps. The expression values were normalized using the DESeq method^68^. Only the conserved clusters were used to compute the eigenvectors using principal component analysis methods. The first and second principal components were visualized in a scatter plot. The normalized gene expression values were also used to compute the standard deviation for each LS-BSR cluster across all samples (excluding isolate 402290) or all isolates (excluding bile samples). The LS-BSR clusters that demonstrated the greatest deviation of expression values were used to generate a clustered heatmap.

All raw data generated by RNA-Seq analysis has been deposited in the short reads archive (SRA) under the accession numbers listed in Table S1, and the expression data is deposited in GEO under the accession number GSE86640.

### EspB Immunoblot assay

Bacteria were inoculated (1:100) from overnight LB cultures into DMEM high glucose or DMEM low glucose media. Cultures were grown to OD_600_ = 1.0. Five ml of culture was pelleted by centrifuging for 10 min at 3,500 x g. Following centrifugation, 1 ml of the supernatant was transferred to a 1.5 μl microcentrifuge tube, and then 5 µl of 5% sodium deoxycholate and 110 µl of ice cold trichloroacetic acid (TCA) (final concentration = 10%) were used to resuspend the pellet. The resuspension was incubated on ice for 15 minutes followed by a 15 min centrifugation at 14,000 × g at 4 °C. The supernatant was removed and the precipitated protein pellet was air-dried and then resuspended in 48 µl of 2X SDS sample loading buffer (Li-Cor) and 12 µl of 2 M Tris base and boiled for 5 min.

The samples were electrophoresed on a 4–15% TGX SDS polyacrylamide gel (BioRad), and transferred to Immobilon PVDF-FL membrane (Millipore). Immunoblot analysis was performed using a chicken directed antibody against EspB (a gift from Dr. J. Kaper) and a donkey anti-chicken IR800 antibody (Li-Cor). Membranes were imaged on a Li-cor Odyssey CLx Infrared Imaging system.

### Immunoblotting of YghJ and the EatA passenger domain

Supernatants of overnight bacterial cultures were precipitated with TCA as above, the resulting pellets were re-suspended in 2X sample buffer separated by SDS-PAGE and transferred to nitrocellulose. After blocking for 1 hour at room temperature with 5% milk in Phosphate Buffered Saline containing 0.005%-Tween 20 (PBS-T) the blots were incubated with rabbit polyclonal antisera against EatA (1:1000 dilution in blocking buffer)^30^ or YghJ^50^ (1:5000 dilution), washed and then probed with Goat anti-rabbit IgG antibody conjugate.

### Heat-labile toxin production

Bacteria were inoculated from −80 °C frozen stock, and grown overnight in casamino acids-yeast extract medium (CAYE) medium at 37 °C with shaking. Overnight cultures were centrifuged at 16,000 rpm for 10 minutes, and the clarified supernatants were used for ELISA (Arbor Assays) as previously described^69^. In brief, 100 µl of culture supernatant was applied to the ELISA plate coated with 0.1 ug of GM1 ganglioside overnight at 4 °C. Plate was incubated at room temperature for 2 hours, then washed with PBS-T (PBS with 0.05% tween 20), followed by blocking with 200 ul of 1% BSA for 1 hour. After blocking, 100 µl of affinity purified anti-LT-B subunit antibody was added in 1:1000 dilution, and incubated for 1 hour at 37 °C. Plates were then washed with PBS-T, followed by the addition of 100 µl of secondary antibody (anti-rabbit-IgG) at a 1:5000 dilution, incubated at 37 °C for 0.5 hours, then washed again with PBS-T. Then 100 µl of TMB substrate mixture was added to each well and the optical density at 650 nm was determined immediately and every 40 seconds thereafter for kinetic analysis. ETEC strains H10407 and jf565 were used as positive and negative controls, respectively.

### Heat-labile toxin delivery assays

The ability of strains to effectively deliver heat-labile toxin to host cells, cultures were grown overnight then used to infect target Caco-2 intestinal epithelial cell monolayers. After incubation for 2.5 hours at 37 °C, 5% CO_2_ supernatant was removed and replaced with pre-warmed tissue culture media, and incubated for another 2 hours. Monolayers were then lysed and cAMP was determined using by ELISA (Arbor Assays).

## Electronic supplementary material

Supplemental Information

11 Supplemental datasets
