# Supplementary material for: Comparative genomics and transcriptomics of Escherichia coli isolates carrying virulence factors of both enteropathogenic and enterotoxigenic E. coli
Source: Sci Rep. 2017 Jun 14;7:3513. doi: 10.1038/s41598-017-03489-z (PMC5471185; doi:10.1038/s41598-017-03489-z)
Supplement: Supplementary file 1 — Supplemental Information [file 41598_2017_3489_MOESM1_ESM.pdf]

Comparative genomics and transcriptomics of *Escherichia coli* isolates carrying virulence factors of both enteropathogenic and enterotoxigenic *E. coli*

Tracy H. Hazen[1,2], Jane Michalski[1], Qingwei Luo[3], Amol C. Shetty[1], Sean C. Daugherty[1], James M. Fleckenstein[3,4], and David A. Rasko[1,2]*

<u>Supplemental Tables</u>

Table S1. Strains analyzed in this study
Table S2. Select LS-BSR gene clusters
Table S3. RNA-Seq samples generated in this study
Table S4. Differential expression of EPEC7 and LT EPEC-specific gene clusters

<u>Supplemental Figures.</u>

Figure S1. Plasmid profile gel
Figure S2. *In silico* detection of *E. coli* virulence genes
Figure S3. Clustered heatmap of p666 compared to all the EPEC and ETEC
Figure S4. Clustered heatmap of p948 compared to all the EPEC and ETEC
Figure S5. FIB *repA* phylogeny
Figure S6. FIIA *repA* phylogeny
Figure S7. PCA plot and heatmap of all samples
Figure S8. ORF diagram, LS-BSR heatmap, and DE heatmap of BFP
Figure S9. Immunoblot analysis of YghJ and EatA
Figure S10. Alignments of *eltA*

<u>Supplemental DataSets</u>

Data Set S1. DE genes of E2348/69 (need to inform journal it is in 0.5 paper)
Data Set S2. DE genes of H10407
Data Set S3. DE genes of HS
Data Set S4. DE genes of 102651
Data Set S5. DE genes of 102712
Data Set S6. DE genes of 401140
Data Set S7. DE genes of 402290 (need to inform journal it is in 0.5 paper)
Data Set S8. LS-BSR values for all ETEC and EPEC in the phylogenomic analysis
Data Set S9. Fasta file of the nucleotide sequences of all LS-BSR gene clusters for all ETEC and EPEC in the phylogenomic analysis (need to add cluster ids to fasta headers)
Data Set S10. LS-BSR values for the ETEC and EPEC isolates analyzed using RNA-Seq
Data Set S11. Fasta file of the nucleotide sequences of all LS-BSR gene clusters for the ETEC and EPEC isolates analyzed using RNA-Seq (need to add cluster ids to fasta headers)

Table S1. Bacterial isolates analyzed in this study

Isolate ID	Pathovar	Phylogroup	AEEC Phylogenomic Lineage	LEE	BFP	LT	ST	EatA	T2SS	Accession No.
EPEC/ETEC										
102651	AEEC	B1	EPEC7	+	-	+	-	-	+	MJIN00000000
102712	AEEC	B1	EPEC7	+	-	+	-	-	+	MJIO00000000
102771	AEEC	B1	EPEC7	+	-	+	-	-	+	MJIP00000000
401140	EPEC	A	EPEC5	+	+	-	-	+	+	JHRM00000000
EPEC										
E2348/69	EPEC	B2	EPEC1	+	+	-	-	-	+	NC_011601.1
2362-75	EPEC	B2	EPEC1	+	+	-	-	-	+	ADUL00000000
B171	EPEC	B1	EPEC2	+	+	-	-	-	-	AAJX00000000.2
32/73	EPEC	B1	EPEC2	+	+	-	-	-	-	LAFA00000000
C581-05	EPEC	B2	EPEC4	+	+	-	-	-	+	AIBE00000000
302150	EPEC	B2	EPEC4	+	+	-	-	-	+	JHSD00000000
103338	EPEC	A	EPEC5	+	+	-	-	-	-	JHSU00000000
402290	EPEC	B1	EPEC7	+	+	-	-	-	+	JHRQ00000000
401091	EPEC	B1	EPEC7	+	+	-	-	-	+	JHSI00000000
400791	EPEC	B1	EPEC7	+	+	-	-	-	+	JHRK00000000
401150	EPEC	B1	EPEC7	+	+	-	-	-	+	JHRN00000000
302687	EPEC	B1	EPEC7	+	+	-	-	-	+	JHSE00000000
302662	EPEC	B1	EPEC7	+	+	-	-	-	+	JHRI00000000
100343	EPEC	B1	EPEC7	+	+	-	-	-	+	JHQW00000000
103573	EPEC	B1	EPEC7	+	+	-	-	-	+	JHRA00000000
200146	EPEC	B1	EPEC7	+	+	-	-	-	+	JHRZ00000000
403341	EPEC	B1	EPEC7	+	-	-	-	-	+	JHTK00000000
303341	EPEC	B1	EPEC7	+	-	-	-	-	+	JHTD00000000
401588	EPEC	B2	EPEC8	+	+	-	-	-	+	JHSK00000000
300214	EPEC	B2	EPEC8	+	+	-	-	-	+	JHSB00000000
302053	EPEC	B2	EPEC9	+	+	-	-	-	+	JHRG00000000
303145	EPEC	B2	EPEC9	+	+	-	-	-	+	JHSG00000000
702324	EPEC	B2	EPEC9	+	+	-	-	-	+	JHRR00000000
100329	EPEC	A	EPEC10	+	+	-	-	-	-	JHRT00000000
C260-92	EPEC	A	EPEC10	+	+	-	-	-	-	AIAO00000000
E110019	AEEC	B1	None	+	-	-	-	-	+	AAJW00000000.2
ETEC										
TW11681	ETEC	A	None	-	-	-	+	+	+	AELD00000000
H10407	ETEC	A	None	-	-	+	+	+	+	FN649414.1
TW10598	ETEC	A	None	-	-	+	+	-	+	AELA00000000
UMNK88	ETEC	A	None	-	-	+	-	-	+	NC_017641.1
2846750	ETEC	A	None	-	-	+	+	+	+	AQGG00000000
MP021566.1	ETEC	A	None	-	-	+	+	+	+	AQEU00000000
BCE019_MS-13	ETEC	A	None	-	-	+	+	+	+	AQCZ00000000
B7A	ETEC	B1	None	-	-	+	+	+	+	AAJT00000000.2
TW14425	ETEC	B1	None	-	-	-	+	-	+	AELE00000000
E24377A	ETEC	B1	None	-	-	+	+	+	+	NC_009801.1
TW10828	ETEC	B1	None	-	-	+	-	-	+	AELC00000000
Jurua 18/11	ETEC	B1	None	-	-	+	-	+	+	AQFB00000000
ThroopD	ETEC	B1	None	-	-	+	+	+	+	AQEJ00000000
2726800	ETEC	B1	None	-	-	+	+	-	+	AQFE00000000
2854350	ETEC	B1	None	-	-	+	+	-	+	APZL00000000
BCE002_MS-12	ETEC	B1	None	-	-	+	-	-	+	AQDA00000000
Envira 8/11	ETEC	B1	None	-	-	+	+	-	+	AQFC00000000

2866350	ETEC	B1	None	-	-	+	+	+	+	APXJ00000000
BCE034_MS-14	ETEC	B1	None	-	-	-	+	+	+	AQCY00000000
2851500	ETEC	B1	None	-	-	+	-	+	+	AQDN00000000
2866450	ETEC	B1	None	-	-	+	-	+	+	AQDI00000000
2845650	ETEC	E	None	-	-	-	[b]+	-	+	AQDR00000000
TW10509	ETEC	F	None	-	-	+	[b]+	+	+	AEHW00000000
Whole-Genome Phylogeny References										
BL21	lab adapted	A	None	-	-	-	-	-	-	NC_012947.1
BW2952	lab adapted	A	None	-	-	-	-	-	-	NC_012759.1
SE11	fecal isolate	B1	None	-	-	-	-	-	+	NC_011415.1
IAI1	fecal isolate	B1	None	-	-	-	-	-	+	NC_011741.1
SMS_3_5	other	F	None	-	-	-	-	-	+	CP000970.1
HS	commensal	A	None	-	-	-	-	-	+	NC_009800.1
ATCC 8739	lab adapted	A	None	-	-	-	-	-	-	NC_010468.1
536	ExPEC	B2	None	-	-	-	-	-	+	NC_008253.1
S88	ExPEC	B2	None	-	-	-	-	-	+	NC_011742.1
UTI89	ExPEC	B2	None	-	-	-	-	-	+	NC_007946.1
CFT073	ExPEC	B2	None	-	-	-	-	-	-	AE014075.1
IA139	ExPEC	F	None	-	-	-	-	-	+	NC_011750.1
UMN026	ExPEC	D	None	-	-	-	-	-	+	NC_011751.1
042	EAEC	D	None	-	-	-	-	-	+	FN554766.1
53638	EIEC	A	None	-	-	-	-	-	+	AAKB00000000.2
55989	EAEC	B1	None	-	-	-	-	-	+	NC_011748.1
TY-2482	EAEC/STEC	B1	None	-	-	-	-	-	+	AFOG00000000.1
S. flexneri 2a 2457T	Shigella	B1	None	-	-	-	-	-	-	NC_004741.1
S. boydii 3083-94	Shigella	B1	None	-	-	-	-	-	+	NC_010658.1
S. sonnei 046	Shigella	B1	None	-	-	-	-	-	-	NC_007384.1
S. dysenteriae 197	Shigella	E	None	-	-	-	-	-	+	NC_007606.1
EDL933	EHEC	E	O157 EHEC1	+	-	-	-	-	-	NC_002655.2
Sakai	EHEC	E	O157 EHEC1	+	-	-	-	-	-	NC_002695.1
CB9615	EPEC	E	O55 AEEC/EHEC1	+	-	-	-	-	+	NC_013941.1
11368	EHEC	B1	non-O157 EHEC2	+	-	-	-	-	-	NC_013361.1
11128	EHEC	B1	non-O157 EHEC2	+	-	-	-	-	+	NC_013364.1

[a]The virulence factor content was determined by *in silico* analysis of the genome assemblies.
[b]A divergent sequence was identified in the genome assembly.

Table S2. Predicted protein functions of select LS-BSR gene clusters

LS-BSR Cluster ID	Gene ID	Predicted Protein Function
In ALL EPEC (LS-BSR ≥0.8) and absent (LS-BSR <0.4) from ALL ETEC:		
Cluster_340		conserved hypothetical protein
Cluster_915		type III secretion apparatus H+-transporting two-sector ATPase
Cluster_3583		type III secretion system regulator family protein
Cluster_4568		type III secretion apparatus protein, YscI/HrpB, C-terminal domain
In ALL ETEC (LS-BSR ≥0.8) and absent (LS-BSR <0.4) from ALL EPEC:		
None		
In ALL EPEC7 (LS-BSR ≥0.8) and absent from ALL other *E. coli* (LS-BSR <0.4):		
Cluster_775		conserved hypothetical protein
In ALL LT+ EPEC7 (LS-BSR ≥0.8) and absent from ALL other *E. coli* (LS-BSR <0.4)		
Cluster_502		conserved hypothetical protein
Cluster_503		conserved hypothetical protein
Cluster_504		putative transcriptional regulator
Cluster_505		AAA domain protein
Cluster_506	*hsdM*	type I restriction-modification system, M subunit
Cluster_625		fimbrial family protein
Cluster_626		hypothetical protein
Cluster_627		helix-turn-helix domain protein
Cluster_629		hypothetical protein
Cluster_630	*tnpA*	transposase for transposon domain protein
Cluster_728		putative prophage protein
Cluster_782		chi7122
Cluster_790		cro/C1-type HTH DNA-binding domain protein
Cluster_791		phage integrase family protein
Cluster_870		TIR domain protein
Cluster_871		conserved hypothetical protein
Cluster_872		RecF/RecN/SMC N terminal domain protein
Cluster_950		hypothetical protein
Cluster_951	*papK*	fimbrial adapter PapK
Cluster_952		helix-turn-helix domain protein
Cluster_1048		conserved hypothetical protein
Cluster_1049	*hsdR*	type I site-specific deoxyribonuclease, HsdR family protein
Cluster_1050		type I restriction modification DNA specificity domain protein
Cluster_1219		putative protein PapJ
Cluster_1252		conserved hypothetical protein
Cluster_1255	*lsoA*	mRNA endoribonuclease LsoA
Cluster_1279		conserved hypothetical protein
In ≥1 EPEC7 (LS-BSR ≥0.8) and absent from all other *E. coli* (LS-BSR <0.4):		
Cluster_775		conserved hypothetical protein
Cluster_3281		hypothetical protein
Cluster_5817		hypothetical protein
Cluster_326		ecoEI R C-terminal family protein
Cluster_327		methyltransferase small domain protein
Cluster_1162		conserved hypothetical protein
Cluster_1163		luxR family transcriptional regulator
Cluster_1619		type I restriction modification DNA specificity domain protein
Cluster_3159		conserved hypothetical protein
Cluster_3160		AAA domain protein

Cluster_3644		conserved hypothetical protein
Cluster_3889	*higA*	addiction module antidote protein, HigA family
Cluster_375		glycosyl transferase family 2 domain protein
Cluster_5017		T5orf172 domain protein
Cluster_943		putative membrane protein
Cluster_945		3-beta hydroxysteroid dehydrogenase/isomerase family protein
Cluster_1225		glycosyl transferase 2 family protein
Cluster_2044		bacterial transferase hexapeptide family protein
Cluster_3925		glycosyl transferases group 1 family protein
Cluster_3926		polysaccharide biosynthesis family protein
Cluster_5747		bacterial transferase hexapeptide family protein
Cluster_3217		conserved hypothetical protein
Cluster_3218		conserved hypothetical protein
Cluster_3236		conserved hypothetical protein
Cluster_3275		conserved hypothetical protein
Cluster_3276		conserved hypothetical protein
Cluster_3440		conserved hypothetical protein
Cluster_3515		conserved hypothetical protein
Cluster_3664		conserved hypothetical protein
Cluster_4223		conserved hypothetical protein
Cluster_5319		conserved hypothetical protein
Cluster_436		acetyltransferase domain protein
Cluster_4222		putative phage superinfection exclusion Cor like protein
Cluster_4825		type I restriction modification DNA specificity domain protein
Cluster_4916		conserved hypothetical protein
Cluster_3489		hypothetical protein
Cluster_5295		AAA domain protein
Cluster_5506		hypothetical protein
Cluster_5294		subtilase family protein
Cluster_502		conserved hypothetical protein
Cluster_503		conserved hypothetical protein
Cluster_504		putative transcriptional regulator
Cluster_505		AAA domain protein
Cluster_506	*hsdM*	type I restriction-modification system, M subunit
Cluster_625		fimbrial family protein
Cluster_626		hypothetical protein
Cluster_627		helix-turn-helix domain protein
Cluster_629		hypothetical protein
Cluster_630	*tnpA*	transposase for transposon domain protein
Cluster_728		putative prophage protein
Cluster_782		putative escherichia coli chi7122 chi7122 genomic chromosome, chi7122
Cluster_790		cro/C1-type HTH DNA-binding domain protein
Cluster_791		phage integrase family protein
Cluster_870		TIR domain protein
Cluster_871		conserved hypothetical protein
Cluster_872		recF/RecN/SMC N terminal domain protein
Cluster_950		hypothetical protein
Cluster_951	*papK*	fimbrial adapter PapK
Cluster_952		helix-turn-helix domain protein
Cluster_1048		conserved hypothetical protein
Cluster_1049	*hsdR*	type I site-specific deoxyribonuclease, HsdR family protein
Cluster_1050		type I restriction modification DNA specificity domain protein
Cluster_1219		putative protein PapJ
Cluster_1252		conserved hypothetical protein

Cluster_1255	*lsoA*	mRNA endoribonuclease LsoA
Cluster_1279		conserved hypothetical protein
Cluster_435		polysaccharide biosynthesis family protein
Cluster_3219		conserved hypothetical protein
Cluster_3993		conserved hypothetical protein
Cluster_5505		glycosyl transferases group 1 family protein
Cluster_711		phosphatase 2C family protein
Cluster_1257		kinase domain protein
Cluster_1258		kinase domain protein
Cluster_964		pentapeptide repeats family protein
Cluster_971		conserved hypothetical protein
Cluster_1951		GDSL-like Lipase/Acylhydrolase family protein
Cluster_2016		tail spike domain protein
Cluster_5690		hypothetical protein
Cluster_221		lacY proton/sugar symporter family protein
Cluster_222		SAF domain protein
Cluster_460		helix-turn-helix domain protein
Cluster_1643		pyridoxal phosphate biosynthetic PdxA family protein
Cluster_1645		bacterial transcriptional regulator family protein
Cluster_1710		NYN domain protein
Cluster_1721		chain length determinant family protein
Cluster_3620		putative escherichia coli chi7122 chi7122 genomic chromosome, chi7122
Cluster_3376		putative membrane protein
Cluster_879		conserved hypothetical protein
Cluster_900		putative domain protein
Cluster_901		P63C domain protein
Cluster_902		conserved hypothetical protein
Cluster_973		transposase, IS605 OrfB family
Cluster_975		hypothetical protein
Cluster_718		initiator Replication family protein
Cluster_1784		hypothetical protein
Cluster_1836		conserved hypothetical protein
Cluster_1851		hypothetical protein
Cluster_1852		hypothetical protein
Cluster_1853		conserved hypothetical protein
Cluster_1854		putative membrane protein
Cluster_1855		hypothetical protein
Cluster_1856		conserved hypothetical protein
Cluster_1857		hypothetical protein
Cluster_1858		hypothetical protein
Cluster_1860		conserved hypothetical protein
Cluster_1865		conserved hypothetical protein
Cluster_1867		HNH endonuclease family protein
Cluster_1868		putative antitermination protein N
Cluster_1871		conserved hypothetical protein
Cluster_1872		dnaB-like helicase N terminal domain protein
Cluster_1873		conserved hypothetical protein
Cluster_1874		hypothetical protein
Cluster_1875		putative membrane protein
Cluster_1876		conserved hypothetical protein
Cluster_1878		conserved hypothetical protein
Cluster_1879		conserved hypothetical protein
Cluster_1882		putative gp68
Cluster_1885		putative protein ninY

Cluster_1886		conserved hypothetical protein
Cluster_1887		hypothetical protein
Cluster_1888		conserved hypothetical protein
Cluster_1889	*19*	lysozyme
Cluster_1891		head fiber family protein
Cluster_1892		hypothetical protein
Cluster_1893		hypothetical protein
Cluster_1895		terminase-like family protein
Cluster_1896		conserved hypothetical protein
Cluster_1897		phage head morphogenesis , SPP1 gp7 family domain protein
Cluster_1898		ADP-ribosyltransferase exoenzyme family protein
Cluster_1899		conserved hypothetical protein
Cluster_1901		putative gp12
Cluster_1902		conserved hypothetical protein
Cluster_1903		conserved hypothetical protein
Cluster_1904		putative gp07
Cluster_1905		conserved hypothetical protein
Cluster_1906		conserved hypothetical protein
Cluster_1907		conserved hypothetical protein
Cluster_1908		conserved hypothetical protein
Cluster_1909		conserved hypothetical protein
Cluster_1910		conserved hypothetical protein
Cluster_1911		conserved hypothetical protein
Cluster_1912		conserved hypothetical protein
Cluster_1913		conserved hypothetical protein
Cluster_1914		putative phage P2 baseplate assembly protein gpV
Cluster_1915		hypothetical protein
Cluster_1916		conserved hypothetical protein
Cluster_1917		putative bacteriophage protein
Cluster_1918		conserved hypothetical protein
Cluster_1919		phage Tail Collar domain protein
Cluster_1920		pectate lyase superfamily protein
Cluster_1921		acyltransferase family protein
Cluster_1966		conserved hypothetical protein
Cluster_1967		reverse transcriptase family protein
Cluster_1988		calcineurin-like phosphoesterase superfamily domain protein
Cluster_1990		HAD hydrolase, IA, variant 1 family protein
Cluster_2008		conserved hypothetical protein
Cluster_2021		yjzC-like family protein
Cluster_2022		conserved hypothetical protein
Cluster_2048		hypothetical protein
Cluster_2051		putative phage protein
Cluster_2077	*toxN*	toxN
Cluster_2080		conserved hypothetical protein
Cluster_3377		glycosyl transferase 2 family protein
Cluster_3911		conserved hypothetical protein
Cluster_3918	*dcm*	DNA (cytosine-5-)-methyltransferase family protein
Cluster_3919		HNH endonuclease family protein
Cluster_3938		winged helix-turn-helix DNA-binding family protein
Cluster_3939		conserved hypothetical protein
Cluster_3942		helix-turn-helix domain protein
Cluster_3943		conserved hypothetical protein
Cluster_3945		phage integrase family protein
Cluster_3968		conserved hypothetical protein

Cluster_3969		helix-turn-helix family protein
Cluster_3970		BRCA1 C Terminus domain protein
Cluster_3971		conserved hypothetical protein
Cluster_3976		conserved hypothetical protein
Cluster_3977		conserved hypothetical protein
Cluster_3978		conserved hypothetical protein
Cluster_3979		conserved hypothetical protein
Cluster_3982		AAA domain protein
Cluster_4023		conserved hypothetical protein
Cluster_4024		conserved hypothetical protein
Cluster_5645		putative membrane protein
Cluster_5646		conserved hypothetical protein
Cluster_5649		RES domain protein
Cluster_5683		conserved hypothetical protein
Cluster_5727		conserved hypothetical protein
Cluster_5768		conserved hypothetical protein
Cluster_5769		conserved hypothetical protein
Cluster_5793		conserved hypothetical protein
In ≥1 LT+ EPEC7 (LS-BSR ≥0.8) and absent from all other *E. coli* (LS-BSR <0.4):		
Cluster_502		conserved hypothetical protein
Cluster_503		conserved hypothetical protein
Cluster_504		putative transcriptional regulator
Cluster_505		AAA domain protein
Cluster_506	*hsdM*	type I restriction-modification system, M subunit
Cluster_625		fimbrial family protein
Cluster_626		hypothetical protein
Cluster_627		helix-turn-helix domain protein
Cluster_629		hypothetical protein
Cluster_630	*tnpA*	transposase for transposon domain protein
Cluster_728		putative prophage protein
Cluster_782		putative escherichia coli chi7122 chi7122 genomic chromosome, chi7122
Cluster_790		cro/C1-type HTH DNA-binding domain protein
Cluster_791		phage integrase family protein
Cluster_870		TIR domain protein
Cluster_871		conserved hypothetical protein
Cluster_872		recF/RecN/SMC N terminal domain protein
Cluster_950		hypothetical protein
Cluster_951	*papK*	fimbrial adapter PapK
Cluster_952		helix-turn-helix domain protein
Cluster_1048		conserved hypothetical protein
Cluster_1049	*hsdR*	type I site-specific deoxyribonuclease, HsdR family protein
Cluster_1050		type I restriction modification DNA specificity domain protein
Cluster_1219		putative protein PapJ
Cluster_1252		conserved hypothetical protein
Cluster_1255	*lsoA*	mRNA endoribonuclease LsoA
Cluster_1279		conserved hypothetical protein

Table S3. RNA-Seq samples generated in this study

Isolate ID	EPEC Phylogenomic Lineage	Phylogroup	Condition[a]	Replicate	Total Reads	Total Reads Mapped	Percent Mapped[b]	SRA Accession No.
E2348/69	EPEC1	B2	LB	1	39,347,430	25,579,676	65	SRX1322473
				2	43,218,880	28,221,680	65	SRX1322474
			LB + bile	1	51,473,692	32,950,310	64	SRS565935
				2	57,686,090	37,569,744	65	SRS565936
			DMEM	1	90,223,668	64,341,630	71	SRX1322475
				2	78,280,450	52,692,826	67	SRX1322476
			DMEM + bile	1	71,198,438	49,864,236	70	SRS565937
				2	57,610,776	41,621,440	72	SRS565938
HS	None		LB	1	50,772,302	32,849,202	65	SRS565948
				2	52,003,340	34,836,598	67	SRS565951
			LB + bile	1	72,007,402	51,786,686	72	SRS565952
				2	72,525,694	53,865,902	74	SRS565953
			DMEM	1	70,158,216	52,992,778	76	SRS565955
				2	74,962,624	57,051,936	76	SRS565957
			DMEM + bile	1	51,096,500	38,117,776	75	SRS565963
				2	62,420,980	48,568,226	78	SRS565969
H10407	None		LB	1	55,498,566	41,800,354	75	SRS565970
				2	51,293,386	38,520,998	75	SRS565976
			LB + bile	1	54,799,240	38,616,902	70	SRS565977
				2	43,799,822	33,340,362	76	SRS565978
			DMEM	1	57,101,468	40,088,102	70	SRS565987
				2	62,931,300	47,969,092	76	SRS565988
			DMEM + bile	1	59,527,736	42,342,120	71	SRS565989
				2	50,365,876	36,116,252	72	SRS566197
102651	EPEC7	B1	LB	1	73,197,712	49,007,590	67	SRS566236
				2	123,721,484	77,226,176	62	SRS566243
			LB + bile	1	49,602,068	31,402,332	63	SRS566244
				2	49,157,028	32,081,032	65	SRS566245
			DMEM	1	27,718,212	18,463,578	67	SRS566265
				2	60,404,908	38,038,916	63	SRS566266
			DMEM + bile	1	59,221,444	38,784,580	65	SRS566268
				2	58,898,112	37,441,562	64	SRS566269
102712	EPEC7	B1	LB	1	57,547,868	36,303,546	63	SRS566161
				2	62,863,746	40,050,722	64	SRS566162
			LB + bile	1	52,634,250	33,158,750	63	SRS566163
				2	53,977,308	32,797,362	61	SRS566164
			DMEM	1	59,134,598	39,267,560	66	SRS566160
				2	58,687,496	37,143,184	63	SRS566213
			DMEM + bile	1	65,860,546	43,115,718	65	SRS566228
				2	61,210,084	40,836,850	67	SRS566235
401140	EPEC5	A	LB	1	98,122,142	62,935,928	64	SRS566270
				2	109,658,950	68,745,070	63	SRS566276
			LB + bile	1	58,269,720	39,640,346	68	SRS567938
				2	64,479,582	44,791,154	69	SRS567940
			DMEM	1	143,164,326	93,428,500	65	SRS566296
				2	68,560,186	43,607,642	64	SRS566297
			DMEM + bile	1	54,837,480	38,290,214	70	SRS567927
				2	64,669,908	43,779,508	68	SRS567937
402290	EPEC7	B1	LB	1	41,672,396	25,888,056	62	SRX481833
				2	38,086,026	23,856,680	63	SRX481834
			DMEM	1	43,796,848	27,200,176	62	SRX481835
				2	57,575,912	38,939,098	68	SRX481836

[a]The *E. coli* isolates were grown to an OD_{600} of 0.5 in either LB broth or DMEM with or without added bile salts.
[b]The percentage of reads mapped includes both genic and intergenic regions.

Table S4. Differential expression of LS-BSR gene clusters that were unique to the EPEC7 and the LT+ EPEC genomes

			LFC[a]												
			H10407				402290	102651				102712			
Cluster ID	Gene ID	Predicted Protein Function	DMEM vs. LB	DMEMB vs. LBB	DMEMB vs. DMEM	LBB vs. LB	DMEM vs. LB	DMEM vs. LB	DMEMB vs. LBB	DMEMB vs. DMEM	LBB vs. LB	DMEM vs. LB	DMEMB vs. LBB	DMEMB vs. DMEM	LBB vs. LB
Gene Clusters of ETEC and LT+ EPEC															
Cluster_166		putative monosaccharide-transporting ATPase	-2.48	NS	NS	NS	NA	NS	NS	NS	NS	NS	-2.08	NS	NS
Cluster_167	*rbsK*	ribokinase	-3.08	-2.52	NS	NS	NA	-3.08	NS	NS	NS	-2.77	-4.24	NS	NS
Cluster_1384		pyocin activator PrtN family protein	NS	NS	NS	NS	NA	NS	NS	NS	NS	NS	-4.52	NS	NS
Cluster_2548	*fucP*	H+ symporter permease	-2.95	NS	NS	NS	NA	-2.23	NS	NS	NS	NS	-2.06	NS	NS
Cluster_3285	*eltB*	heat-labile enterotoxin beta chain family protein	NS	-3.24	-2.17	NS	NA	-3.97	NS	NS	NS	NS	-4.27	-2.31	NS
Cluster_3286	*eltA*	heat-labile enterotoxin alpha chain family protein	-2.19	-2.54	NS	NS	NA	-3.32	NS	NS	NS	NS	-4.26	NS	NS
Gene Clusters of EPEC7															
Cluster_2234		binding-protein-dependent transport system inner membrane component family protein	NA	NA	NA	NA	NS	-2.62	NS	NS	NS	NS	-4.75	NS	NS
Cluster_2596		hypothetical protein	NA	NA	NA	NA	NS	NS	NS	NS	NS	NS	-4.32	NS	NS
Cluster_2876		helix-turn-helix domain protein	NA	NA	NA	NA	NS	NS	NS	NS	NS	NS	-2.92	NS	NS
Cluster_3059	*bor*	lipoprotein Bor	NA	NA	NA	NA	-3.81	-2.99	NS	2.35	NS	-3.20	-2.49	NS	NS
Cluster_4588		polysaccharide deacetylase family protein	NA	NA	NA	NA	-3.61	-3.17	-2.62	2.15	NS	-3.88	-2.43	2.36	NS
Cluster_4588		polysaccharide deacetylase family protein	NA	NA	NA	NA	NS	-3.17	-2.62	2.15	NS	-3.88	-2.43	2.36	NS
Cluster_4702		alpha amylase, N-terminal ig-like domain protein	NA	NA	NA	NA	NS	NS	NS	NS	NS	NS	-2.65	NS	NS
Cluster_4704		bacterial extracellular solute-binding family protein	NA	NA	NA	NA	NS	-2.38	NS	NS	NS	NS	-4.22	NS	NS
Cluster_4705		putative CymA protein	NA	NA	NA	NA	NS	-3.35	NS	NS	NS	NS	NS	NS	NS
Cluster_5167		conserved hypothetical protein	NA	NA	NA	NA	NS	NS	NS	NS	NS	NS	-5.61	-4.37	NS
Cluster_593		conserved hypothetical protein	NA	NA	NA	NA	-3.64	-3.61	-2.35	2.35	NS	-4.41	-3.19	NS	NS
Cluster_596		glycosyl transferases group 1 family protein	NA	NA	NA	NA	-3.19	-3.22	NS	2.09	NS	-4.24	-2.64	NS	NS
Cluster_901		repair family protein	NA	NA	NA	NA	NS	NS	NS	NS	NS	NS	-2.98	-2.77	NS
Gene Clusters of LT+ EPEC															
Cluster_1479		PLD-like domain protein	NA	NA	NA	NA	NA	-4.63	-3.13	NS	NS	-3.93	-4.18	NS	NS
Cluster_1806		putative membrane protein	NA	NA	NA	NA	NA	-2.43	NS	NS	NS	NS	-3.67	NS	NS
Cluster_1927	*papA*	Pap fimbrial major pilin protein	NA	NA	NA	NA	NA	NS	NS	4.50	5.42	NS	-2.21	4.22	5.75
Cluster_1931		helix-turn-helix domain protein	NA	NA	NA	NA	NA	NS	NS	3.20	5.33	NS	-3.30	2.65	5.61
Cluster_1934		PapG chaperone-binding domain protein	NA	NA	NA	NA	NA	NS	NS	2.34	3.19	NS	NS	2.21	4.02
Cluster_26		aspartate racemase family protein	NA	NA	NA	NA	NA	NS	NS	NS	NS	NS	NS	3.88	NS
Cluster_3228	*lsoB*	antitoxin LsoB	NA	NA	NA	NA	NA	NS	NS	NS	NS	NS	-2.03	NS	NS
Cluster_329	*papH*	PAP fimbrial minor pilin protein	NA	NA	NA	NA	NA	NS	NS	3.46	NS	NS	-2.58	NS	3.08
Cluster_3432		conserved hypothetical protein	NA	NA	NA	NA	NA	-3.23	NS	NS	NS	NS	NS	NS	NS
Cluster_3459		TIR domain protein	NA	NA	NA	NA	NA	-2.40	NS	NS	NS	-2.66	-3.05	NS	NS
Cluster_81		aminotransferase class I and II family protein	NA	NA	NA	NA	NA	-2.01	NS	NS	NS	NS	NS	NS	NS

[a]LFC is the log_2 fold-change

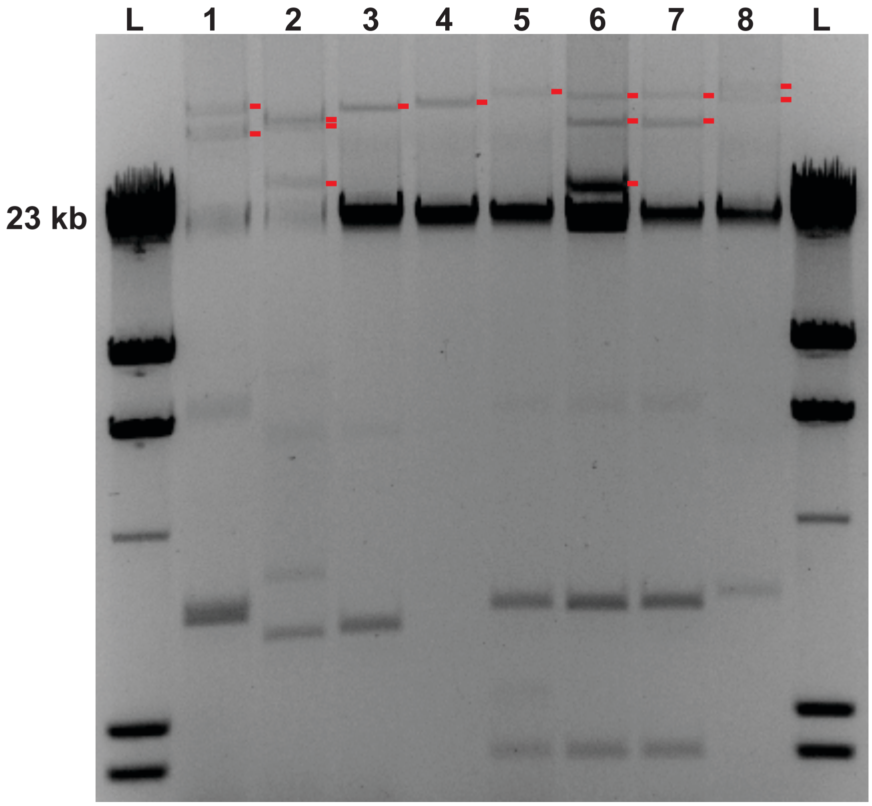

Figure S1. Plasmid profiles of the EPEC/ETEC isolates. Agarose gel electrophoresis of plasmids in ETEC isolates H10407 (1) and E24377A (2), tEPEC isolates E2348/69 (3) and 402290 (4), and EPEC/ETEC hybrid isolates 102651 (5), 102712 (6), 102771 (7), and 401140 (8) with the lambda DNA Hind-III digested ladder (NEB) (L). Each of the large plasmid bands (>23 kb) is indicated by a red line. The top band of the lambda DNA ladder is approximately 23 kb and corresponds with the sheared linearized chromosomal DNA present in each of the plasmid extractions.

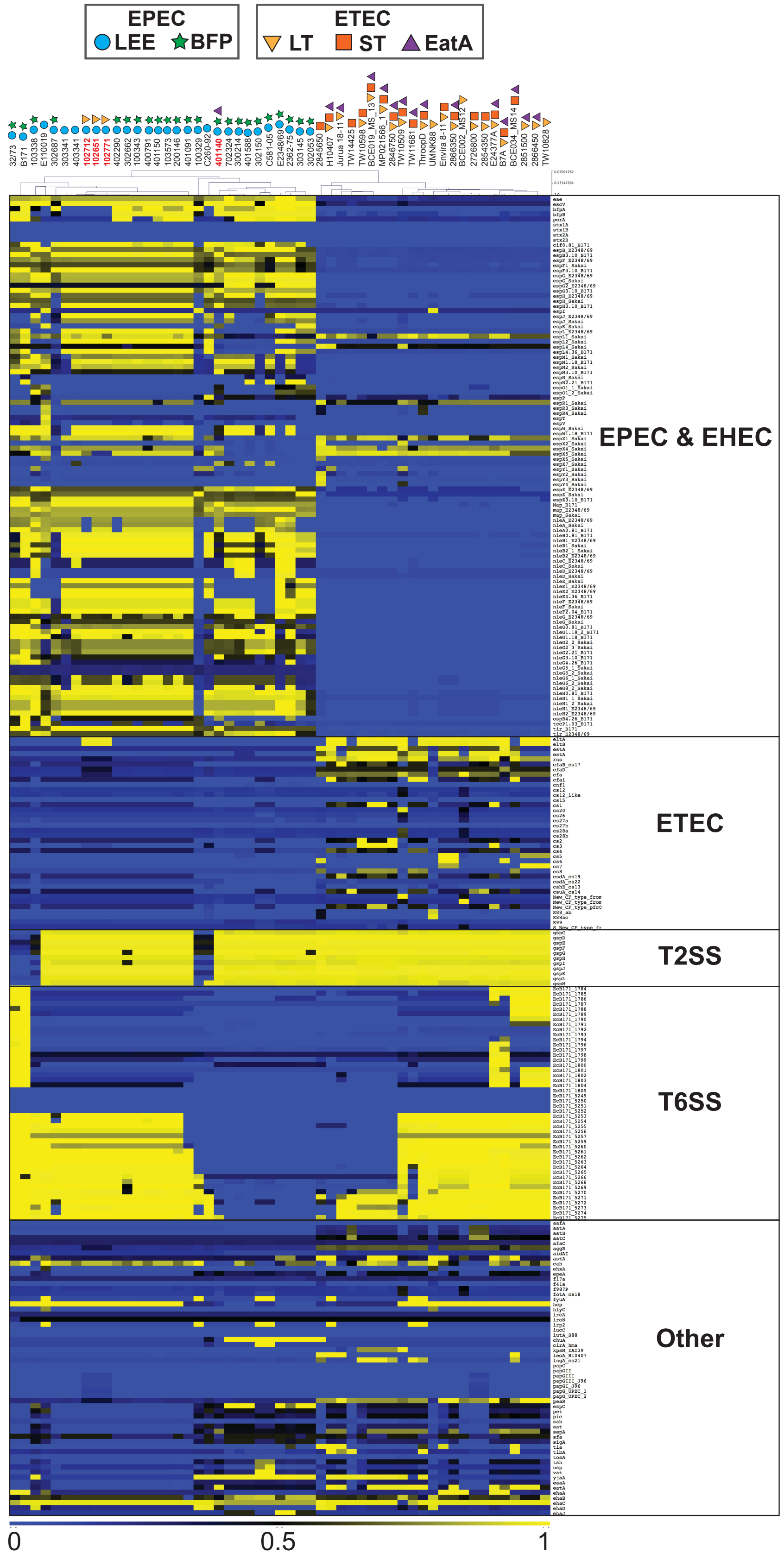

EPEC
LEE
BFP
ETEC
LT
ST
EatA
EPEC & EHEC
ETEC
T2SS
T6SS
Other
0
0.5
1

Figure S2. ***In silico*** **detection of** ***E. coli*** **virulence genes.** The protein-encoding regions of previously described *E. coli* virulence genes were identified in each of the EPEC and ETEC genomes using TBLASTN LS-BSR [1,2] and visualized using MEV[3]. Each row represents a virulence gene and each column is a different genome. The genomes were grouped by hierarchical cluster analysis of their LS-BSR values, with yellow indicating the presence and blue indicating the absence of each virulence gene in the genomes. The EPEC/ETEC hybrid isolates are indicated in bold red.

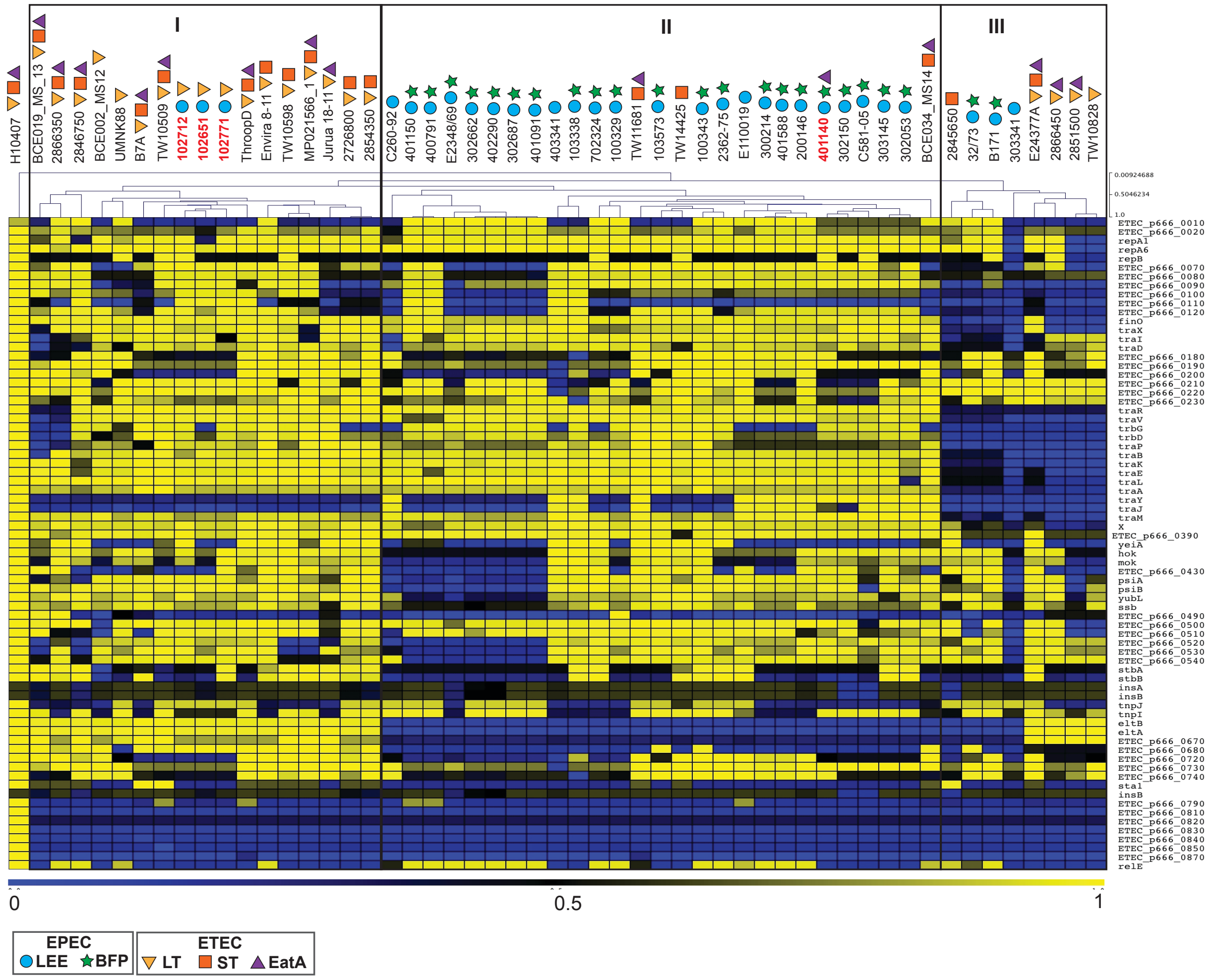

Figure S3. *In silico* detection of an LT-encoding plasmid, p666. The protein-encoding genes of plasmid p666 from ETEC isolate H10407[4] were identified in each of the EPEC and ETEC genomes using TBLASTN LS-BSR[1,2]. Each row represents a plasmid gene and each column is a different genome. The genomes were grouped by hierarchical cluster analysis of their LS-BSR values using MEV[3]. Yellow indicates the presence and blue indicates the absence of each plasmid gene in the genomes. The EPEC/ETEC isolates are indicated in bold red.

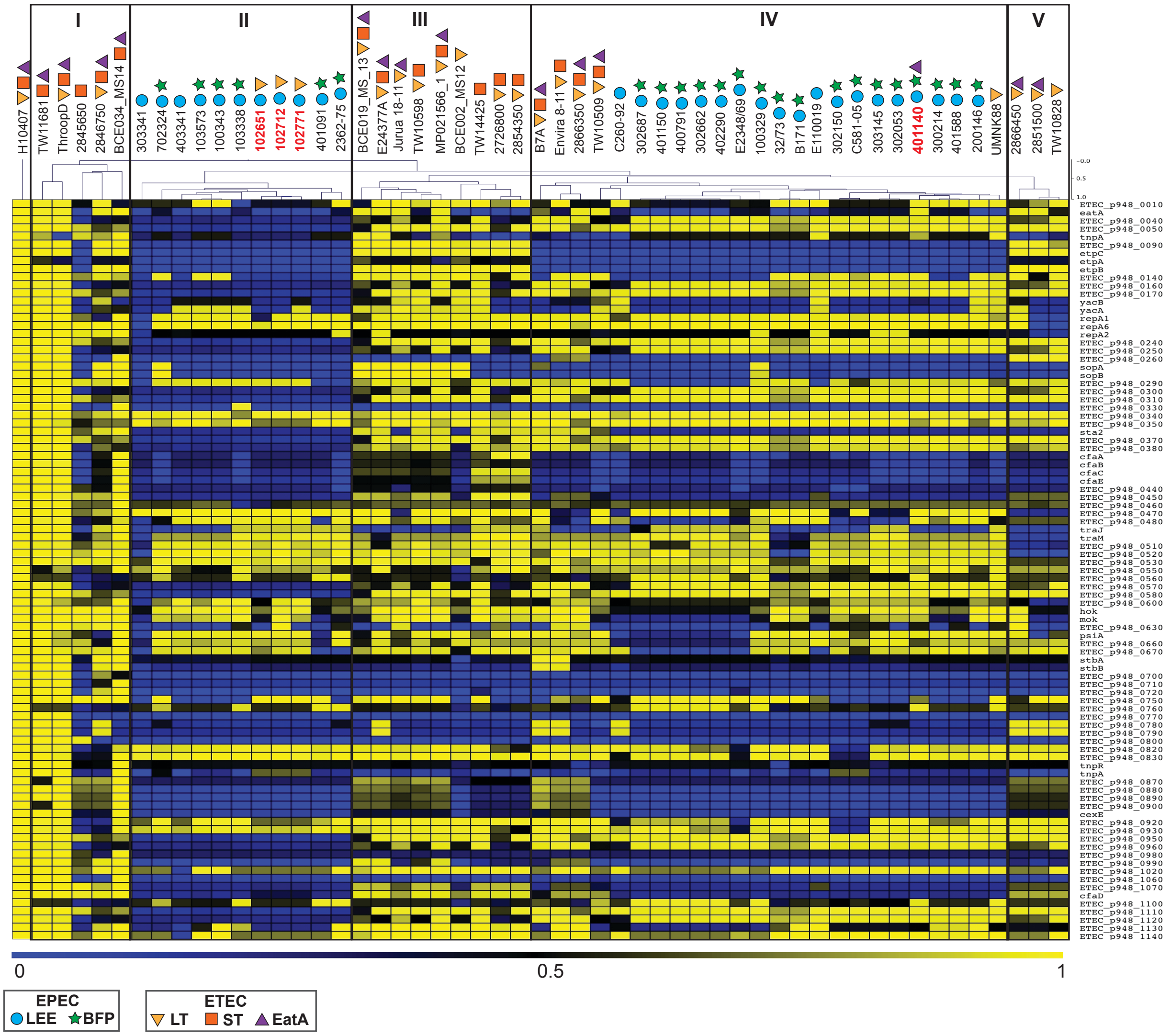

Figure S4. *In silico* detection of an EatA-encoding plasmid, p948. The protein-encoding genes of plasmid p948 from ETEC isolate H10407[4] were identified in each of the EPEC and ETEC genomes using TBLASTN LS-BSR[1,2]. Each row represents a plasmid gene and each column is a different genome. The genomes were grouped by hierarchical cluster analysis of their LS-BSR values using MEV [3], with yellow indicating the presence and blue indicating the absence of each plasmid gene in the genomes. The EPEC/ETEC isolates are indicated in bold red.

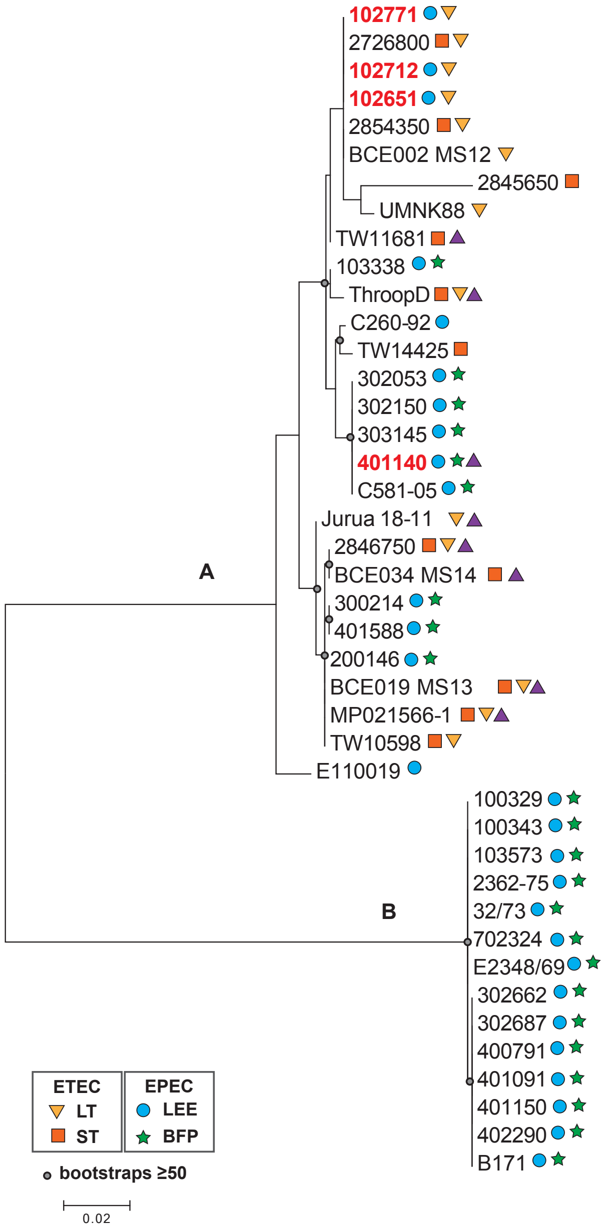

Figure S5. FIB *repA* phylogeny. A maximum-likelihood phylogeny comparing the nucleotide sequences of the putative FIB-type *repA* genes identified in the EPEC and ETEC genomes analyzed in this study including the EPEC/ETEC hybrid isolates. The maximum-likelihood phylogeny was generated using the Kimura 2-parameter model and 1,000 bootstrap values with MEGA6 [5]. Virulence genes present in each of the genome sequences are indicated by symbols as follows: LT (yellow triangle), ST (orange

square), LEE (blue circle), BFP (green star), and EatA (purple triangle). The EPEC/ETEC isolates are indicated in bold red and all fall into group A. The scale bar indicates the distance of 0.02 nucleotide substitutions per site. The grey circles on the nodes of the tree indicate a bootstrap value of ≥50.

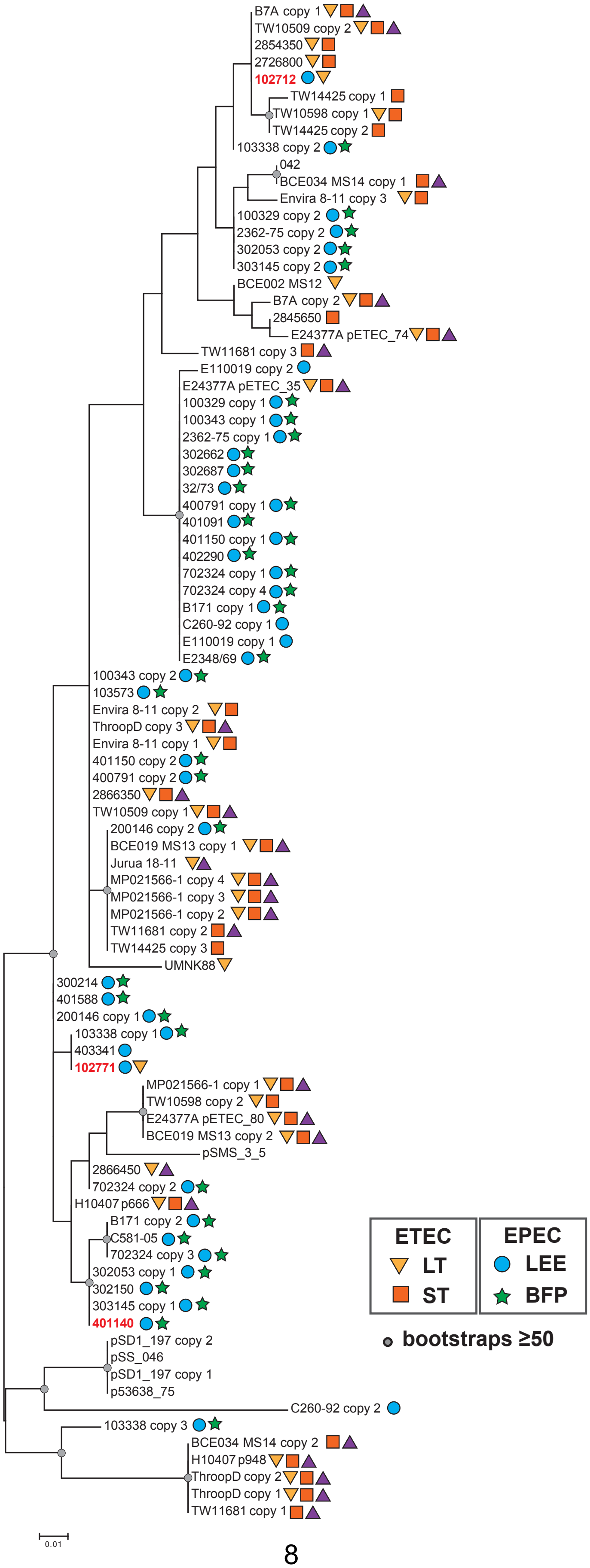
B7A copy 1
TW10509 copy 2
2854350
2726800
102712
TW14425 copy 1
TW10598 copy 1
TW14425 copy 2
103338 copy 2
042
BCE034 MS14 copy 1
Envira 8-11 copy 3
100329 copy 2
2362-75 copy 2
302053 copy 2
303145 copy 2
BCE002 MS12
B7A copy 2
2845650
E24377A pETEC_74
TW11681 copy 3
E110019 copy 2
E24377A pETEC_35
100329 copy 1
100343 copy 1
2362-75 copy 1
302662
302687
32/73
400791 copy 1
401091
401150 copy 1
402290
702324 copy 1
702324 copy 4
B171 copy 1
C260-92 copy 1
E110019 copy 1
E2348/69
100343 copy 2
103573
Envira 8-11 copy 2
ThroopD copy 3
Envira 8-11 copy 1
401150 copy 2
400791 copy 2
2866350
TW10509 copy 1
200146 copy 2
BCE019 MS13 copy 1
Jurua 18-11
MP021566-1 copy 4
MP021566-1 copy 3
MP021566-1 copy 2
TW11681 copy 2
TW14425 copy 3
UMNK88
300214
401588
200146 copy 1
103338 copy 1
403341
102771
MP021566-1 copy 1
TW10598 copy 2
E24377A pETEC_80
BCE019 MS13 copy 2
pSMS_3_5
2866450
702324 copy 2
H10407 p666
B171 copy 2
C581-05
702324 copy 3
302053 copy 1
302150
303145 copy 1
401140
pSD1_197 copy 2
pSS_046
pSD1_197 copy 1
p53638_75
C260-92 copy 2
103338 copy 3
BCE034 MS14 copy 2
H10407 p948
ThroopD copy 2
ThroopD copy 1
TW11681 copy 1
0.01
ETEC
LT
ST
EPEC
LEE
BFP
bootstraps ≥50

Figure S6. FIIA *repA* phylogeny. A maximum-likelihood phylogeny comparing the nucleotide sequences of the putative FIIA-type *repA* genes identified in the EPEC and ETEC genomes analyzed in this study including the EPEC/ETEC hybrid isolates. The maximum-likelihood phylogeny was generated using the Kimura 2-parameter model and 1,000 bootstrap values with MEGA6 [5]. Virulence genes present in each of the genome sequences are indicated by symbols as follows: LT (yellow triangle), ST (orange square), LEE (blue circle), BFP (green star), and EatA (purple triangle). The EPEC/ETEC hybrid isolates are indicated in bold red. The scale bar indicates the distance of 0.01 nucleotide substitutions per site. The grey circles on the nodes of the tree indicate a bootstrap value of ≥50%.

A.

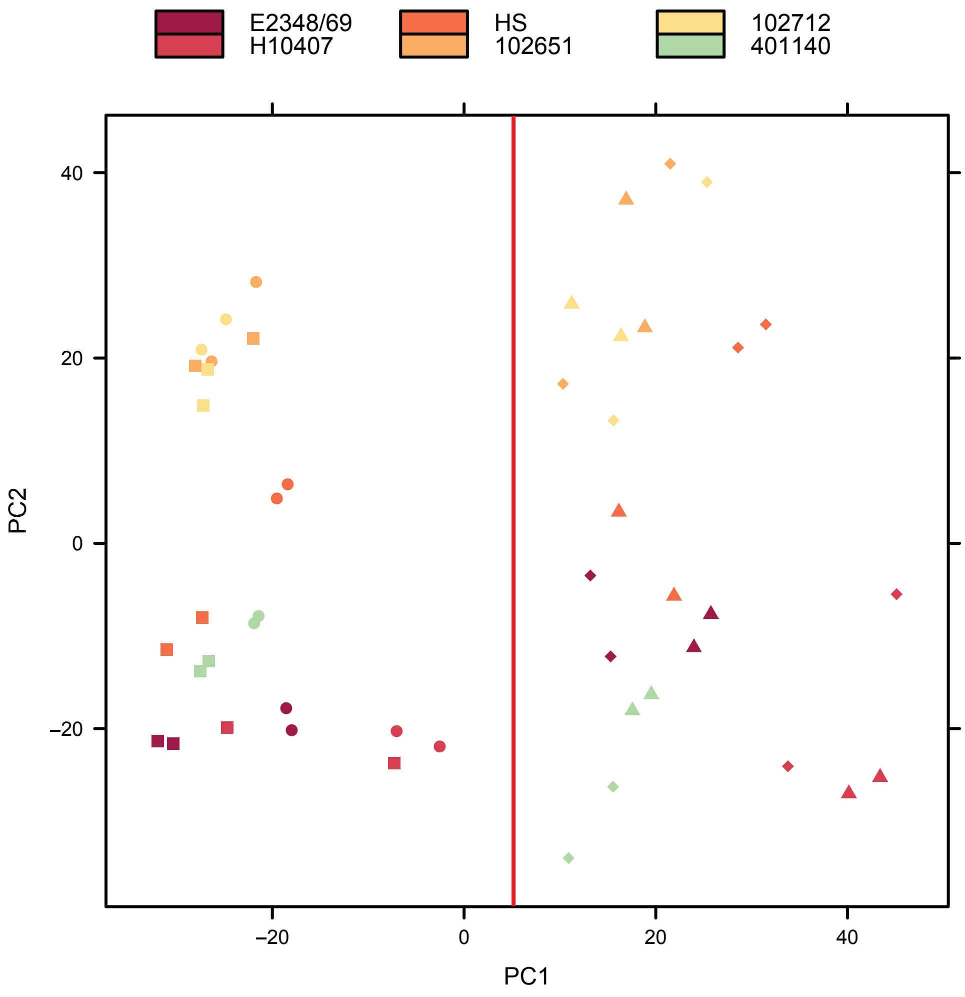
E2348/69
H10407
HS
102651
102712
401140
PC2
40
20
0
−20
PC1
−20
0
20
40

B.

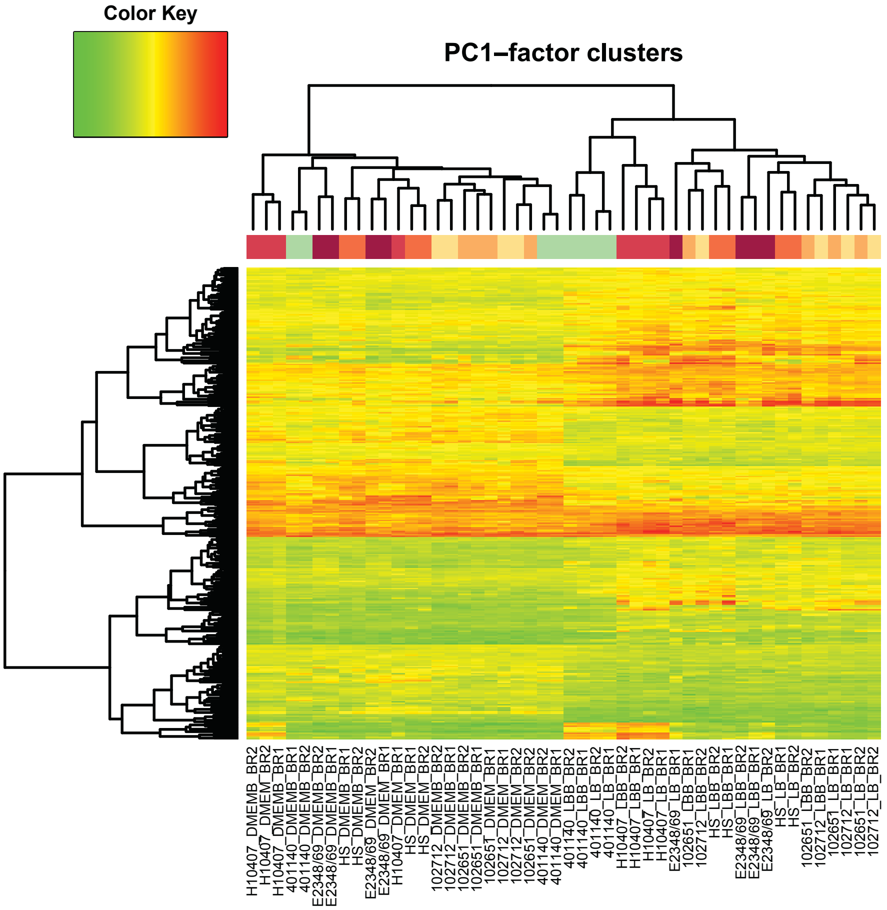
Color Key
PC1–factor clusters
H10407_DMEMB_BR2
H10407_DMEM_BR2
H10407_DMEMB_BR1
401140_DMEMB_BR1
401140_DMEMB_BR2
E2348/69_DMEMB_BR2
E2348/69_DMEMB_BR1
HS_DMEMB_BR1
HS_DMEMB_BR2
E2348/69_DMEM_BR2
E2348/69_DMEM_BR1
H10407_DMEM_BR1
HS_DMEM_BR1
HS_DMEM_BR2
102712_DMEMB_BR2
102712_DMEMB_BR1
102651_DMEMB_BR2
102651_DMEMB_BR1
102651_DMEM_BR1
102712_DMEM_BR1
102712_DMEM_BR2
102651_DMEM_BR2
401140_DMEM_BR2
401140_DMEM_BR1
401140_LBB_BR2
401140_LBB_BR1
401140_LB_BR2
401140_LB_BR1
H10407_LBB_BR2
H10407_LBB_BR1
H10407_LB_BR2
H10407_LB_BR1
E2348/69_LB_BR1
102651_LBB_BR1
102712_LBB_BR2
HS_LBB_BR2
HS_LBB_BR1
E2348/69_LBB_BR2
E2348/69_LBB_BR1
E2348/69_LB_BR2
HS_LB_BR1
HS_LB_BR2
102651_LBB_BR2
102712_LBB_BR1
102651_LB_BR1
102712_LB_BR1
102651_LB_BR2
102712_LB_BR2

C.

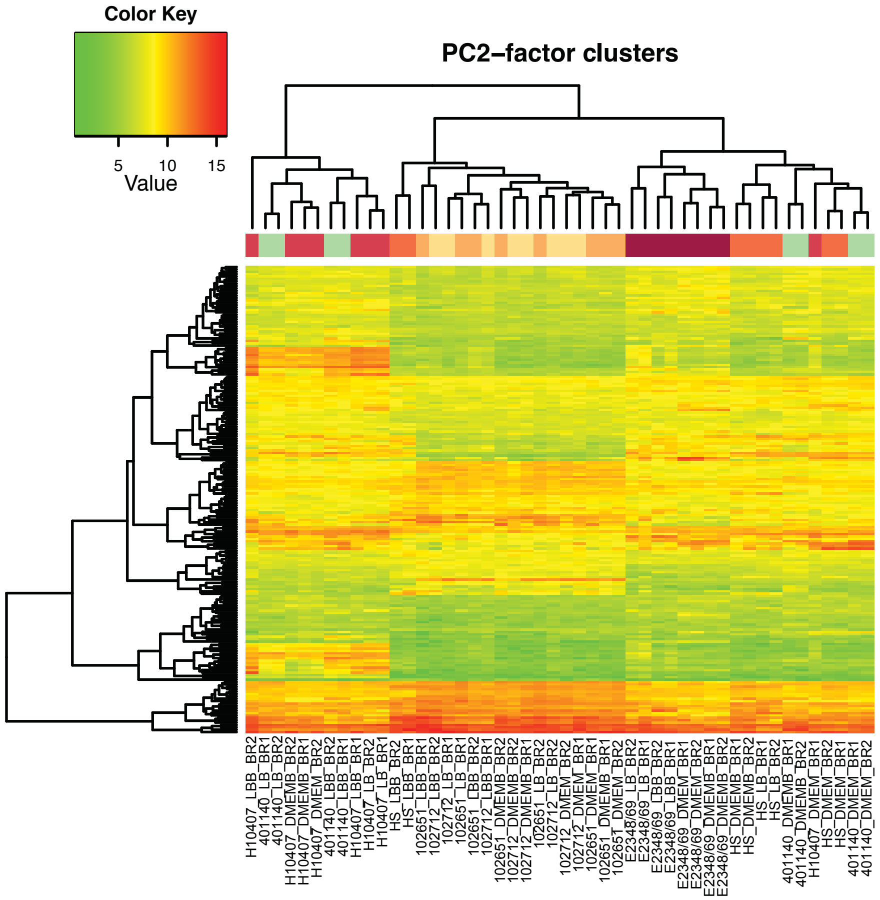

Figure S7. Analysis of the RNA-Seq samples with and without bile. A) Principal component analysis of the normalized expression values of the RNA-Seq samples with (LBB and DMEMB) and without (LB and DMEM) bile for ETEC isolate H10407, non-pathogenic isolate HS, EPEC isolate E2348/69, and the EPEC/ETEC isolates 102651, 102712, and 401140. Each of the RNA-Seq samples is indicated by a symbol as follows: LB (triangle), LBB (diamond), DMEM (square), and DMEMB (circle). Each of the *E. coli* isolates is identified by a different color. The red line separates the LB and DMEM samples. Biological replicates cluster together in this analysis. **B)** Hierarchical clustering of 333 gene clusters of PC1 and **C)** 196 gene clusters of PC2. The heatmap displays the normalized gene expression values of the LS-BSR gene clusters that were present in all of the genomes and had the greatest deviation in their expression values.

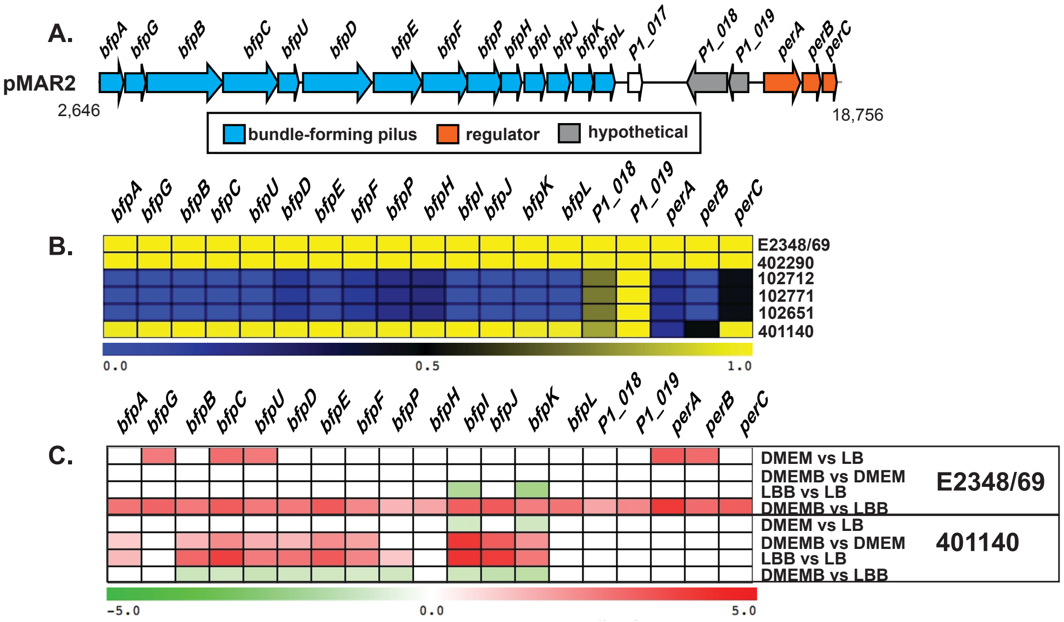

Figure S8. Differential expression of protein-encoding genes within the BFP region. A) Diagram of the protein-encoding genes within the BFP region of the sequenced EPEC plasmid, pMAR2, of EPEC isolate E2348/69. The predicted protein function is indicated by the color of the arrow, and the size and direction of the arrows indicates the size of each predicted gene and the direction of transcription. **B)** *In silico* detection of the protein-encoding genes of the BFP region of pMAR2 in each of the EPEC genomes analyzed in this study. The colors of the heatmap represent the BSR values of each gene, with yellow indicating a gene is present and blue indicating a gene is absent. **C)** Heatmap of the differential expression for each of the sample comparisons of EPEC isolates E2348/69 and 401140, which contain the BFP region. Only significant $\log_2$ fold-change (LFC) values ≥1 or ≤-1 are included in the heatmap. Red indicates increased differential expression while green indicates decreased

differential expression. White indicates that a gene was either not present in the EPEC isolate, and/or did not exhibit significant differential expression.

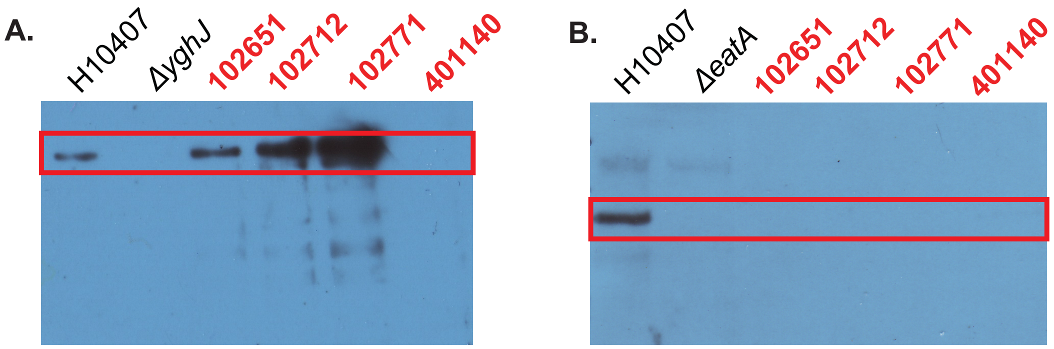

Figure S9. Immunoblot analysis of secreted proteins for YghJ and EatA. A) Detection of YghJ, a metalloprotease secreted by the T2SS, and **B)** the autotransporter protein EatA by immunoblot as previously described in Luo *et al*. [6] and Patel *et al*. [7], respectively. The EPEC/ETEC hybrid isolates are indicated in bold red and a red box is drawn around the expected location of the protein band relative to the positive control strain H10407. All EPEC/ETEC hybrids, with the exception of 401140 secreted YghJ, whereas none of the hybrid isolates secreted EatA.

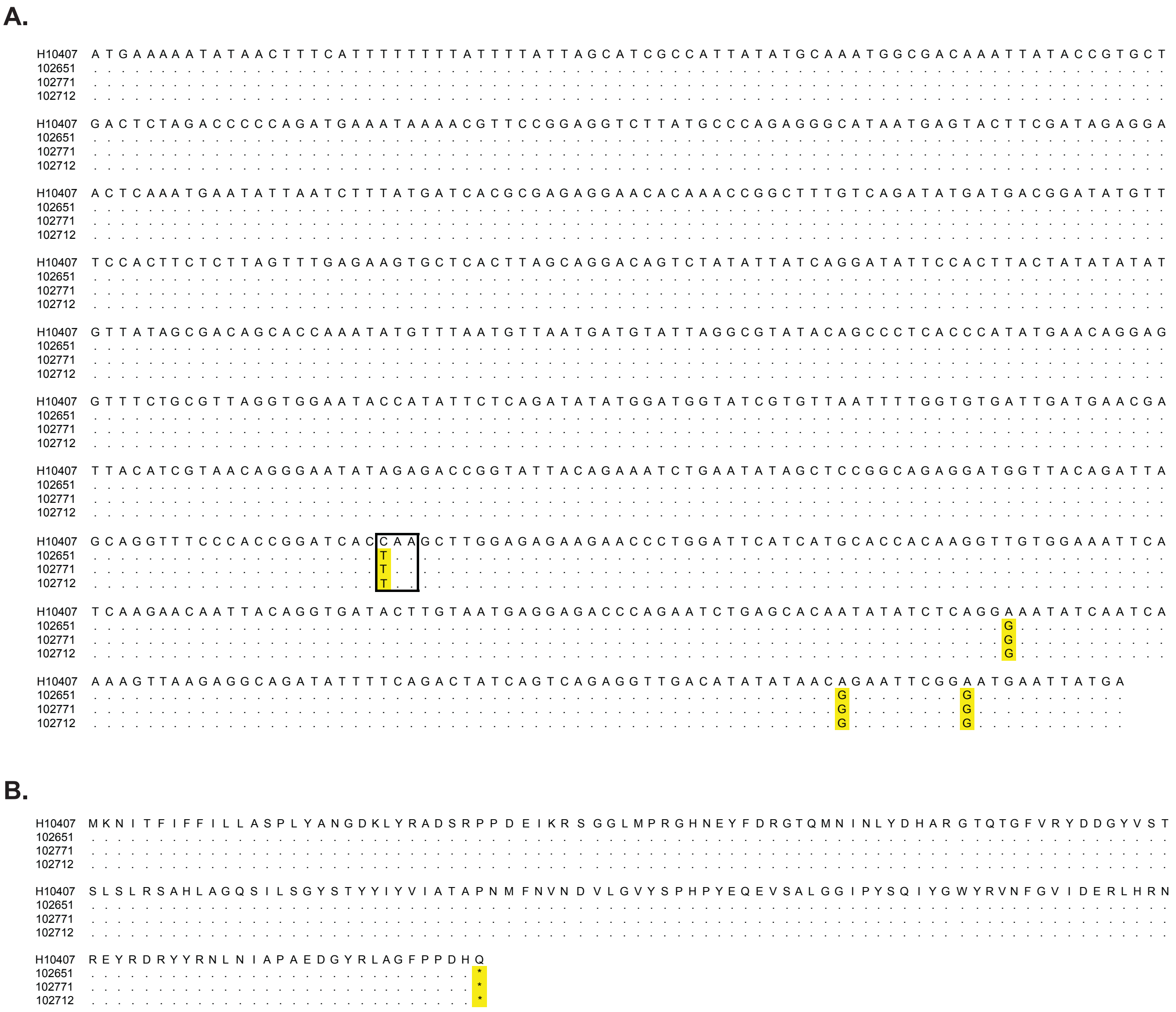

Figure S10. Alignment of *eltA*. The predicted EltA-encoding region of the EPEC/ETEC hybrids 102651, 102712, and 102771 were aligned to the *eltA* gene of the ETEC reference strain H10407. The nucleotide sequences are aligned in panel A, while the predicted amino acid sequences are aligned in panel B. The alignments were generated using ClustalW of MEGA6 [8]. Similarities are indicated with a dot while nucleotide or amino acid differences are shown and highlighted in yellow. A box is drawn around the nucleotide change that resulted in the introduction of a stop codon and truncation of the coding region in the predicted *eltA* gene of the EPEC/ETEC hybrids.

References

1 Sahl, J. W., Caporaso, J. G., Rasko, D. A. & Keim, P. The large-scale blast score ratio (LS-BSR) pipeline: a method to rapidly compare genetic content between bacterial genomes. *PeerJ* **2**, e332, doi:10.7717/peerj.332 (2014).

2 Hazen, T. H. *et al.* Refining the pathovar paradigm via phylogenomics of the attaching and effacing *Escherichia coli*. *Proc Natl Acad Sci USA* **110**, 12810-12815, doi:10.1073/pnas.1306836110 (2013).

3 Saeed, A. I. *et al.* TM4 microarray software suite. *Methods Enzymol* **411**, 134-193, doi:10.1016/S0076-6879(06)11009-5 (2006).

4 Crossman, L. C. *et al.* A commensal gone bad: complete genome sequence of the prototypical enterotoxigenic *Escherichia coli* strain H10407. *J Bacteriol* **192**, 5822-5831, doi:10.1128/JB.00710-10 (2010).

5 Tamura, K., Stecher, G., Peterson, D., Filipski, A. & Kumar, S. MEGA6: Molecular Evolutionary Genetics Analysis version 6.0. *Mol Biol Evol* **30**, 2725-2729, doi:10.1093/molbev/mst197 (2013).

6 Luo, Q. *et al.* Enterotoxigenic *Escherichia coli* secretes a highly conserved mucin-degrading metalloprotease to effectively engage intestinal epithelial cells. *Infect Immun* **82**, 509-521, doi:10.1128/IAI.01106-13 (2014).

7 Patel, S. K., Dotson, J., Allen, K. P. & Fleckenstein, J. M. Identification and molecular characterization of EatA, an autotransporter protein of enterotoxigenic *Escherichia coli*. *Infect Immun* **72**, 1786-1794 (2004).

8 Kumar, S., Stecher, G. & Tamura, K. MEGA7: Molecular Evolutionary Genetics Analysis Version 7.0 for Bigger Datasets. *Mol Biol Evol* **33**, 1870-1874, doi:10.1093/molbev/msw054 (2016).
